# Jagged1 regulates extracellular matrix deposition and remodeling in triple-negative breast cancer

**DOI:** 10.1126/sciadv.aea9562

**Published:** 2026-03-18

**Authors:** Marjaana Parikainen, Ujjwal Suwal, Pekka Rappu, Jyrki Heino, Cecilia M. Sahlgren

**Affiliations:** ^1^ Faculty of Science and Engineering, Cell Biology, Åbo Akademi University, Turku, Finland.; ^2^ InFLAMES Research Flagship, Åbo Akademi University and University of Turku, Turku, Finland.; ^3^ Turku Bioscience Center, Åbo Akademi University and University of Turku, Turku, Finland.; ^4^ Department of Life Technologies, University of Turku, Turku, Finland.; ^5^ Department of Biomedical Engineering, Eindhoven University of Technology, Eindhoven, Netherlands.; ^6^ Institute for Complex Molecular Systems (ICMS), Eindhoven University of Technology, Eindhoven, Netherlands.

## Abstract

The extracellular matrix (ECM) and tumor microenvironment heterogeneity drive cancer progression and treatment resistance. High Jagged1 expression correlates with poor patient survival and promotes tumor growth and invasion in triple-negative breast cancer (TNBC). Using transcriptomics, proteomics, and imaging of cancer cell/fibroblast cocultures in vitro and in vivo, we demonstrate that Jagged1-mediated cross-talk between TNBC cells and fibroblasts enhances myofibroblast activation, collagen accumulation, and alignment of ECM fibers. In single-cell RNA sequencing data of TNBC tumors, high Jagged1 expression gives rise to a myofibroblast subpopulation previously associated with enhanced invasion. Jagged1 increases transforming growth factor–β (TGFβ) activity in fibroblast cocultures, and TGFβ inhibition prevents the Jagged1-induced ECM alignment. Thus, Jagged1 regulates ECM remodeling upstream of TGFβ. Furthermore, higher substrate stiffness up-regulates Jagged1, suggesting a feed-forward loop between Jagged1, ECM stiffness, and TGFβ. With the emergence of safe therapeutics targeting specific Notch components, Jagged1 modulation may offer an approach for treating invasive breast cancer.

## Teaser

Jagged1 drives malignant ECM remodeling and tumor microenvironment heterogeneity via increased TGFβ activity in fibroblasts.

## INTRODUCTION

Tumor progression and metastasis depend on the cancer cell–intrinsic changes and the formation of a supportive tumor microenvironment (TME) (*1*, *2*). The TME consists of various types of nonmalignant cells, including immune cells, endothelial cells, adipocytes, and fibroblasts, surrounded by the extracellular matrix (ECM). The ECM is a complex and dynamic network of cross-linked proteins that coordinates cellular behavior in multicellular organisms. Increased matrix deposition, stiffness, and a more aligned ECM organization, stimulated by such key signaling pathways as transforming growth factor–β (TGFβ) and hypoxia signaling, drive tumor cell malignancy and metastasis (*3*–*5*). The stiff ECM of tumors is increasingly recognized as a major factor hindering immune infiltration and promoting therapy resistance, and thus, understanding the mechanisms driving ECM dysregulation in the TME is of utmost importance for the development of novel cancer therapeutics.

In malignant tumors, cancer-associated fibroblasts (CAFs) play a critical role in synthesis and organization of the ECM. Diverse cellular origin, spatial location, and inherent plasticity of the CAFs make them a versatile cell type for which three major phenotypes have been described: myofibroblastic CAFs (myCAFs), inflammatory and growth factor–enriched CAFs (iCAFs), and antigen-presenting CAFs (apCAFs) (*6*). myCAFs actively secrete ECM proteins, such as fibronectin (FN) and collagens, and ECM-modifying enzymes, including matrix metalloproteases (MMPs) and lysyl oxidase (LOX) (*6*–*9*). myCAFs are typically contractile and express α-smooth muscle actin (αSMA). In different tumors, myCAFs may either prevent or promote cancer progression. Breast cancer is the leading cause of cancer death in women, complicated by molecular subtypes defined by estrogen receptor (ER), progesterone receptor (PR), and human epidermal growth factor receptor 2 (HER2) status. Triple-negative breast cancer (ER−/PR−/HER2−) is aggressive and treatment-resistant, underscoring the need for new therapies. In breast cancer, the abundance of αSMA-positive myCAFs correlates with a more aggressive phenotype and disease relapse (*10*, *11*). Multiple paracrine factors within the TME have been reported to induce the differentiation of stromal fibroblasts or iCAFs into myCAFs, especially TGFβ. Emerging evidence suggests that juxtacrine cell-cell contact–mediated mechanisms may also play a role in this context (*12*–*17*).

The Notch signaling pathway has a critical role in embryogenesis and maintenance of most tissues, and aberrant Notch signaling is frequently observed in various cancers. Notch signaling is activated when a Notch ligand interacts with a Notch receptor in a cell-cell contact–dependent manner. Upon activation, the receptor undergoes two subsequent cleavages to release its intracellular domain, which then translocates to the nucleus to activate target gene expression. Mammalian cells express four Notch receptors (Notch1 to Notch4) and five Notch ligands (Jagged1, Jagged2, Dll1, Dll3, and Dll4). Notch activating cell-cell contacts in cancer may occur either between cancer cells or cancer cells and cells of the TME. Both oncogenic and tumor-suppressive effects of Notch have been shown, depending on the context, and Notch signaling is involved in all hallmarks of cancer (*18*).

In breast cancer, the Notch signaling pathway has been implicated in tumor progression and triple-negative breast cancer (TNBC) (*19*). Most research has focused on the role of Notch receptors in breast cancer pathogenesis, while comprehensive studies on the functional roles of Notch ligands are lagging behind. The Notch ligand Jagged1 is overexpressed in several cancer types and participates in many tumor-promoting activities, such as increased proliferation, vascularization, and invasion (*20*). In breast cancer, high Jagged1 expression has been associated with the aggressive basal subtype and an increased risk of metastasis (*21*–*24*), but a more detailed understanding of Jagged1-mediated tumorigenic functions is still missing.

In this study, we show that Jagged1 promotes the in vivo growth and invasion of TNBC cells. High expression of Jagged1 predicts poor patient survival, specifically in aggressive breast cancer subtypes (HER2-enriched and basal-like/TNBC). Using previously published single-cell RNA sequencing (scRNA-seq) data (*25*), publicly available data of patients with breast cancer (*26*), and a three-dimensional (3D) in vitro model, we demonstrate that Jagged1 enhances invasiveness, cancer stem cell (CSC)–like features, and expression of ECM-related genes in TNBC cells. High Jagged1 in cancer cells correlates with an increased differentiation of myCAFs with a Notch and TGFβ gene signature in scRNA-seq data of TNBC tumors. When Jagged1-expressing cancer cells were cocultured with fibroblasts, collagen production and ECM fiber alignment were highly increased in a TGFβ-dependent manner. Furthermore, increasing substrate stiffness elevates Jagged1 expression levels in cancer cells, pointing toward a feed-forward loop between tumor-promoting ECM remodeling and Jagged1. Last, we validate the found relationship between Jagged1, TGFβ, myCAF activation, and increased collagen deposition using in vivo coculture tumors and datasets of patients with breast cancer. Together, our data designate Jagged1 as a central regulator of TGFβ activity and ECM remodeling in TNBC, thus promoting cancer progression.

## RESULTS

### Jagged1 enhances the aggressive behavior of TNBC cells in vivo

**Fig. 1.**
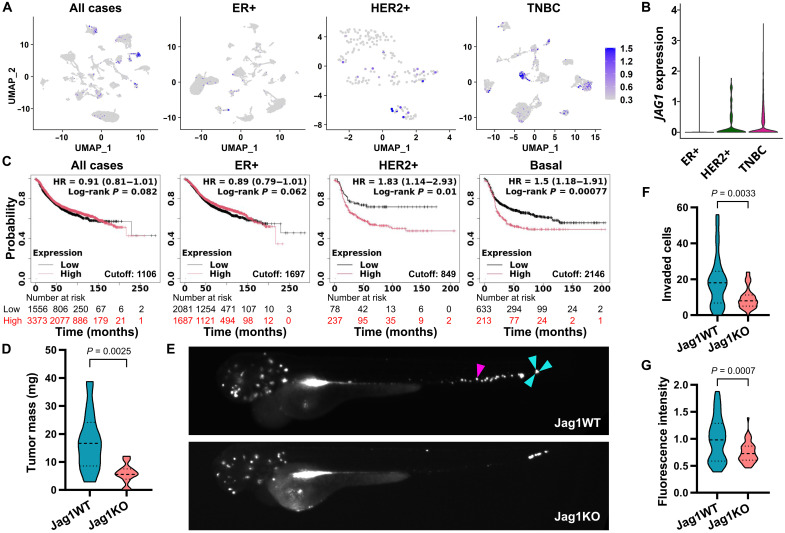
Jagged1 promotes breast cancer aggressiveness. (**A** and **B**) *JAG1* expression in scRNA-seq data (*25*) of cancer cells from different subtypes of breast cancer. *n* = 15,816 cells from 31 patients in all cases, *n* = 10,427 cells from 15 patients in ER+, *n* = 176 cells from 3 patients in HER2+, and *n* = 5213 cells from 13 patients in TNBC. UMAP, uniform manifold approximation projection. (**C**) Relapse-free survival of patients with a low (black) or high (red) expression of Jag1 in different subtypes of breast cancer in the Kaplan-Meier Plotter database (*58*). HR, hazard ratio. (**D**) Tumor masses of MDA-MB-231 WT (Jag1WT) and Jag1 knockout (Jag1KO) cell chick CAM xenograft models. *P* values are calculated by a two-tailed unpaired *t* test. *n* = 10 tumors for Jag1WT and *n* = 12 for Jag1KO. (**E**) Representative images of zebrafish embryos injected intravenously with fluorescently labeled cancer cells. The magenta arrowhead denotes an example of an individual cancer cell in the tail of the embryo, and cyan arrowheads mark a cluster of three cells. (**F**) Quantification of cells that have invaded into the tails of zebrafish embryos. The *P* value is calculated by a two-tailed unpaired Mann-Whitney test. *n* = 30 embryos for Jag1WT and *n* = 42 for Jag1KO. (**G**) Normalized fluorescence intensity of cells invaded to the heads of zebrafish embryos. The *P* value is calculated by a two-tailed unpaired *t* test. *n* = 30 embryos for Jag1WT and *n* = 44 for Jag1KO.

To investigate how Jagged1 affects breast cancer progression, we first assessed Jagged1 expression levels in scRNA-seq data (*25*) of cancer cells from different subtypes of breast cancer. To exclude any effect caused by immunotherapy, we analyzed only treatment-naïve samples. Jagged1 was more highly expressed in the aggressive breast cancer subtypes (HER2-enriched and TNBC) while being expressed at a low level in ER+ cancer cells (Fig. 1, A and B). By using published patient datasets (*26*), we found that high Jagged1 expression was associated with worse relapse-free survival in the same aggressive subtypes where it is more highly expressed (Fig. 1C). High Jagged1 expression has been previously linked to ER− breast cancer (*21*, *27*, *28*). We also confirmed that Jagged1 was highly expressed in a panel of ER− breast cancer cell lines and low in ER+ cell lines (fig. S1A). High Jagged1 expression led to worse survival in a cohort of patients with ER− breast cancer while having the opposite effect in ER+ breast cancer (fig. S1B), further supporting the notion that Jagged1 drives breast cancer progression in the absence of estrogen stimulation. To clarify the role of Jagged1 in aggressive breast cancer, we generated a Jag1 CRISPR-Cas9 knockout cell line out of MDA-MB-231 human TNBC cells (Jag1KO). First, we assessed the ability of Jag1KO cells to form tumors in vivo in the chick chorioallantoic membrane (CAM) model in comparison to the parental MDA-MB-231 wild-type (WT) cell line, which retains a high Jagged1 expression (Jag1WT). Loss of Jagged1 led to a substantial decrease in tumor growth (Fig. 1D). The average mass of Jag1WT tumors was 17.1 mg, whereas Jag1KO tumors were only 5.8 mg. In addition, when injected intravenously into zebrafish embryos, Jag1KO cells showed decreased survival in the circulation and reduced formation of metastases (Fig. 1, E to G). The amount of individual Jag1KO cells in zebrafish embryo tails was 57% lower than for Jag1WT cells, whereas the fluorescence intensity of cell clusters in zebrafish embryo heads was 25% lower for Jag1KO cells. Together, our data show that Jagged1 increases TNBC cell growth and invasion in vivo and is associated with poor prognosis in aggressive, ER− breast cancer.

### Jagged1 regulates the TNBC cell phenotype and gene expression

**Fig. 2.**
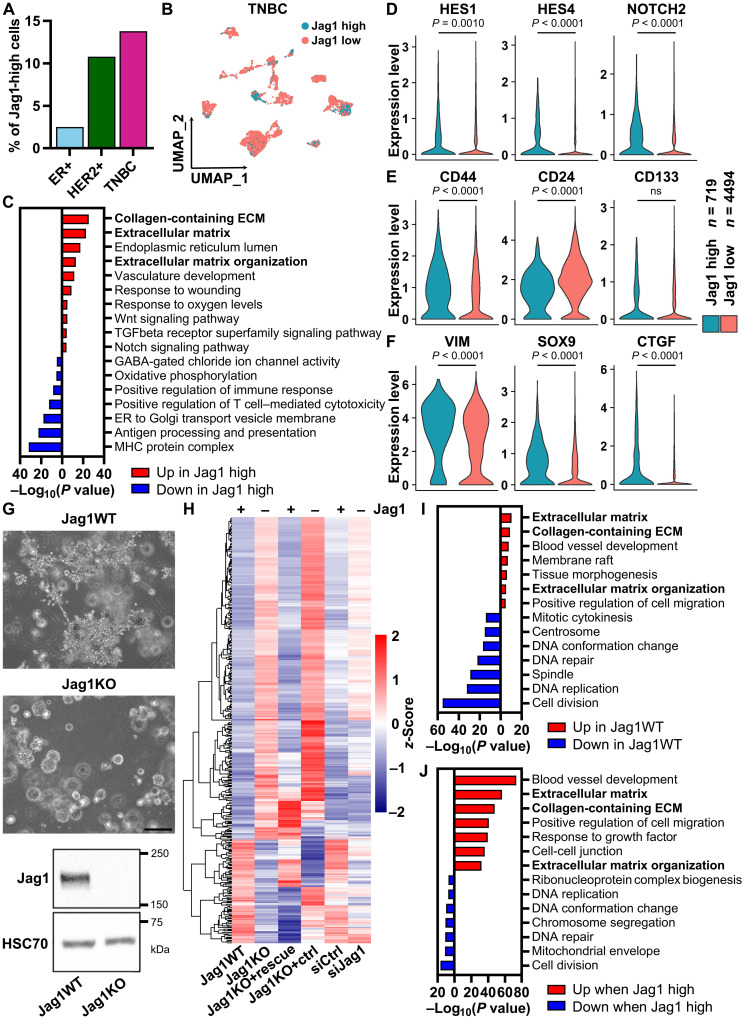
Jagged1 enhances invasiveness, CSC-like features, and expression of ECM genes in TNBC cells. (**A**) Percentage of cancer cells with high *JAG1* expression in scRNA-seq data (*25*) of different breast cancer subtypes. (**B**) UMAP visualization of TNBC cells in the scRNA-seq data split into Jag1-high and Jag1-low populations on the basis of the *JAG1* expression level. *n* = 719 Jag1-high TNBC cells and *n* = 4494 Jag1-low TNBC cells. (**C**) GSEA of genes differentially expressed between Jag1-high and Jag1-low TNBC cells in scRNA-seq data (fdr < 0.01 and average log_2_ fold change >0.500). Examples of (**D**) Notch pathway genes, (**E**) breast CSC markers, and (**F**) EMT markers differentially expressed between Jag1-high and Jag1-low TNBC cells in scRNA-seq data. *P* values are calculated by a Wilcoxon rank sum test with Benjamini-Hochberg adjustment. ns, not significant. (**G**) Representative images of MDA-MB-231 Jag1WT and Jag1KO 3D spheroids and a Western blot showing Jag1 expression in the cell lines. Scale bar, 200 μm. (**H**) Clustered heatmap of a genome-wide transcriptome analysis showing *z*-scores of genes differentially expressed between spheroid samples of Jag1WT and Jag1KO cells, Jag1KO cells transfected with Jag1-containing plasmid (Jag1KO + rescue) or an empty vector plasmid (Jag1KO + ctrl), and Jag1WT cells transfected with nontargeting siRNA (siCtrl) or Jag1-targeting siRNA (siJag1) (fdr < 0.05 and fold change >1.5). *n* = 3 biological replicates. (**I**) GSEA of genes differentially expressed between Jag1WT and Jag1KO spheroids (fdr < 0.05). (**J**) GSEA of genes either positively (red) or negatively (blue) correlating with JAG1 expression (Spearman’s correlation coefficient >0.300 or <−0.300, respectively) in an mRNA expression dataset of patients with breast cancer (TCGA, Cell 2015, *n* = 817) (*26*).

To clarify the mechanisms behind Jagged1-mediated TNBC progression, we split the cancer cells of the scRNA-seq dataset (*25*) into cells with either high (Jag1-high) or low (Jag1-low) expression of *JAG1*. TNBC tumors had the highest percentage of Jag1-high cells (Fig. 2, A and B). A gene set enrichment analysis (GSEA) of genes differentially expressed between Jag1-high and Jag1-low cells (table S1) revealed ECM-related genes as the most significantly up-regulated genes in Jag1-high cells (Fig. 2C). In addition, Jag1-high cells displayed increased expression of genes involved in the Notch, Wnt, and TGFβ pathways, along with increased expression of breast CSC and epithelial-mesenchymal transition (EMT) markers (Fig. 2, C to F). We set up 3D Matrigel spheroid cultures of MDA-MB-231 cells to assess the impact of Jagged1 on growth and invasiveness and to elucidate the Jagged1-driven transcriptome. After 7 days of culture, Jag1KO spheroids were less invasive and smaller compared to Jag1WT spheroids and appeared more differentiated (Fig. 2G). A similar phenotype was seen with small interfering RNA (siRNA)–mediated silencing of Jagged1, while reintroducing Jagged1 into Jag1KO cells saved the phenotype (fig. S2A). The effect seemed to be Notch signaling–dependent, as inhibition of Notch by using a gamma-secretase inhibitor or knocking out Notch1 induced a similar phenotype (fig. S2B). A genome-wide transcriptome analysis of these spheroid samples revealed hundreds of genes differentially expressed depending on the Jagged1 expression status (Fig. 2H and table S2). We validated the RNA sequencing results of a few hit genes by quantitative polymerase chain reaction (PCR) (fig. S2C). A GSEA showed that ECM- and cell migration–related gene ontology terms were significantly overrepresented among genes up-regulated in Jag1WT cells (Fig. 2I). Several collagens, integrins, and matrix-remodeling enzymes were found among the Jagged1-regulated ECM genes (table S2). Notably, highly similar gene sets were correlated with the Jagged1 expression level in a human database of patients with breast cancer (Fig. 2J and table S3) (*26*). In conclusion, higher Jagged1 expression correlated with increased CSC-like features and invasiveness, both in vitro and in vivo, in data of patients with breast cancer.

### Jagged1 induces ECM deposition and remodeling in fibroblast cocultures via TGFβ signaling

**Fig. 3.**
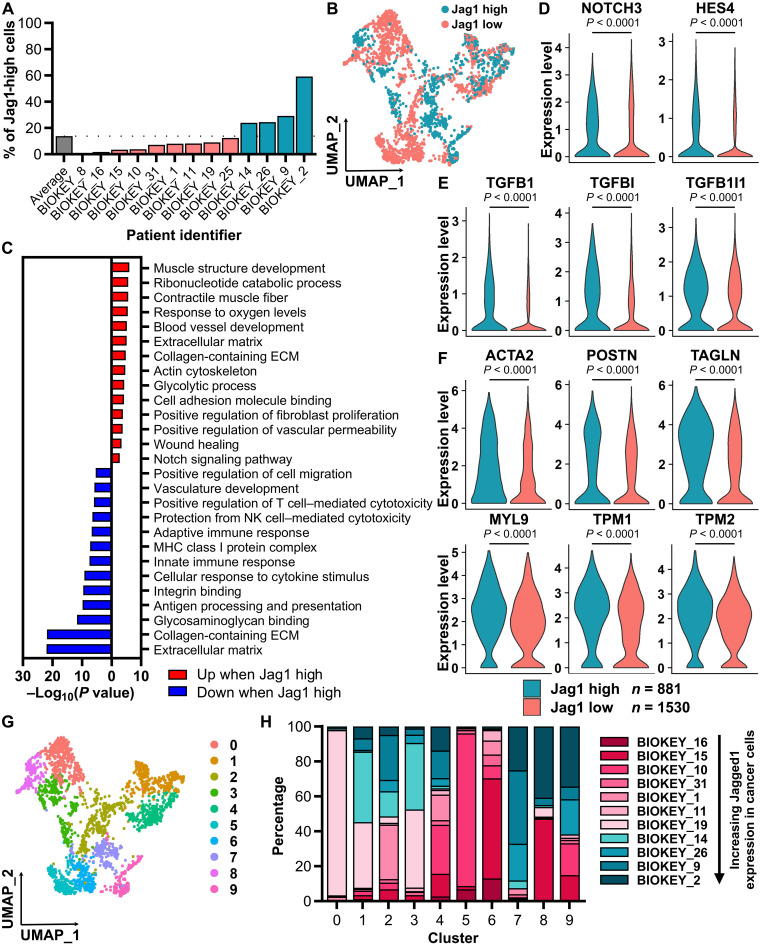
High Jagged1 in cancer cells promotes Notch and TGFβ pathways and cellular contractility in fibroblasts. (**A**) Percentage of cancer cells with high *JAG1* expression in scRNA-seq data (*25*) of TNBC samples from individual patients. (**B**) UMAP visualization of fibroblasts from patients with either high or low Jag1 in cancer cells. *n* = 881 of Jag1-high fibroblasts and *n* = 1530 of Jag1-low fibroblasts. (**C**) GSEA of genes differentially expressed between fibroblasts from patients with high or low Jag1 in cancer cells (fdr < 0.01 and average log_2_ fold change >0.500). Expression of (**D**) Notch pathway genes, (**E**) TGFβ pathway genes, and (**F**) myofibroblast markers in fibroblasts from patients with high or low Jag1 in cancer cells. *P* values are calculated by a Wilcoxon rank sum test with Benjamini-Hochberg adjustment. (**G**) UMAP visualization of fibroblast clusters. (**H**) Percentage of fibroblasts from each individual patient in fibroblast clusters.

ECM components can, in principle, be produced by any cell, and it has been shown that cancer cells can have an altered expression of, for example, different collagens and matrix-remodeling enzymes (*29*). However, the major ECM producers are fibroblasts and, especially in the context of cancer, the myCAFs. Given that Jagged1 up-regulated the expression of ECM-related genes in cancer cells, we assessed how Jagged1 expressed by cancer cells affects ECM production by fibroblasts. For this, we first checked the abundance of Jag1-high cancer cells in individual patients with TNBC in the scRNA-seq data and then split patients into Jag1-high or Jag1-low groups on the basis of whether they had a higher or lower percentage of Jag1-high cells than the average (Fig. 3, A and B). We then performed a GSEA of genes differentially expressed in fibroblasts from Jag1-high and Jag1-low patients (table S4). Fibroblasts from tumors with high *JAG1* expression in cancer cells showed increased expression of Notch target genes, a higher proportion of Notch3-positive cells, and an increased expression of *TGFB1* and other TGFβ pathway genes, as well as myCAF and cellular contractility markers (Fig. 3, C to F), while the expression of several iCAF markers was down-regulated (fig. S3A).

In contrast to the up-regulation of ECM-related genes observed in Jag1-high cancer cells, there was a Jagged1-dependent shift in the composition of the expressed ECM genes in fibroblasts, with some genes being up-regulated and others down-regulated (fig. S3, B and C). There were no significant differences in the expression of fibrillar collagens, while several TGFβ-regulated collagen fiber–modifying enzymes were up-regulated in Jag1-high fibroblasts (fig. S3D). As we saw an increase in the expression of myCAF markers and a decrease in iCAF markers in Jag1-high fibroblasts, we hypothesized that Jagged1 induces a phenotypic switch of the fibroblasts and thus affects their clustering in the scRNA-seq data. When we clustered the fibroblasts, we noticed one cluster consisting almost entirely of fibroblasts from tumors with high Jagged1 in cancer cells (Fig. 3, G and H). This fibroblast cluster exhibited highly up-regulated Notch and TGFβ pathway signatures, as well as increased expression of myCAF markers, fibrillar collagens, and ECM-modifying enzymes (fig. S4, A to E, and table S5). The gene markers of this cluster showed high similarity to the markers of the specific myCAF subpopulation, TGFβ-myCAFs, previously found by Kieffer *et al.* (*30*), while *JAG1* expression also correlated significantly with the expression of TGFβ-myCAF markers in data of patients with breast cancer (fig. S4F). This suggests that Jagged1 induces activation of this specific myCAF subpopulation in breast cancer. To verify that the Jagged1-induced myofibroblast marker expression is not due to an increased amount of other cell types, such as smooth muscle cells, mistakenly classified as fibroblasts in the scRNA-seq data and enriched in the Jag1-high tumors, we checked the expression levels of smooth muscle cell, pericyte, adipocyte, and fibroblast markers in the fibroblast clusters (fig. S5, A to D). None of the clusters stood out as positive for smooth muscle cell markers, whereas some clusters showed positivity for pericyte or adipocyte markers. This can point to the origin of fibroblasts in those specific clusters or indicate the presence of other cell types besides fibroblasts. The presence of contractile, αSMA-expressing pericytes in the fibroblast population could skew our conclusions on myCAF differentiation. However, when we removed the pericyte marker–positive clusters from the fibroblast population and reanalyzed the data, there was still an increase in Notch and TGFβ pathway signatures and myCAF markers in fibroblasts from Jag1-high tumors (fig. S5, E to G, and table S6). In addition, there was one cluster in the reanalyzed fibroblasts consisting almost entirely of Jag1-high tumor fibroblasts that expressed markers of TGFβ-myCAFs, such as *LAMP5* (fig. S5F and table S7), further validating the involvement of Jagged1 in inducing this specific fibroblast subpopulation.

**Fig. 4.**
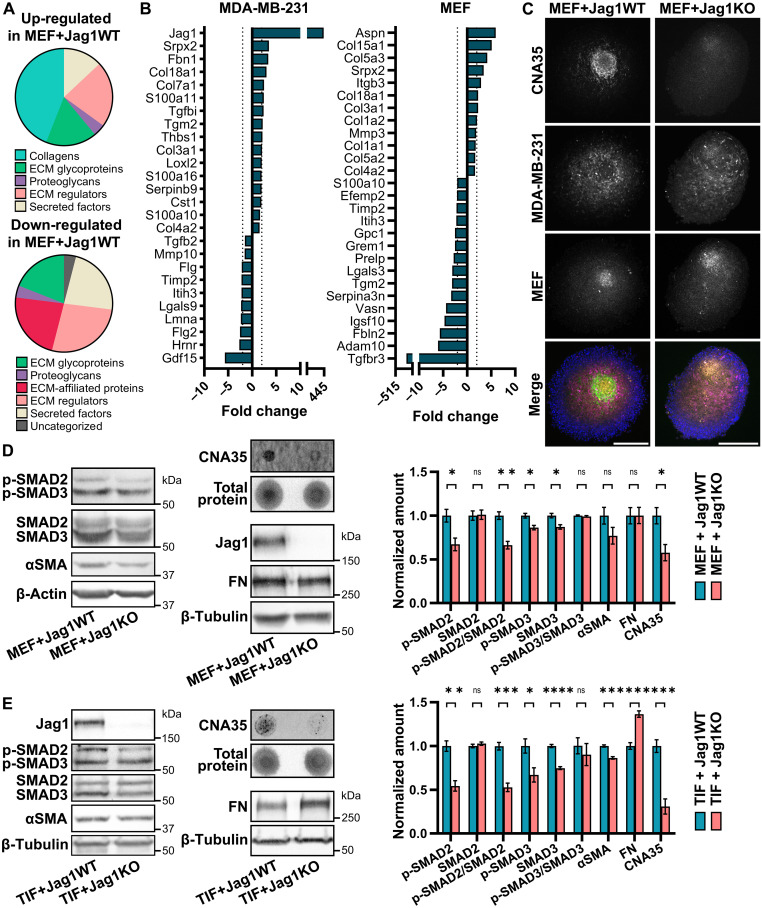
Jagged1 signaling from cancer cells induces fibroblast activation, collagen production, and TGFβ signaling in 3D cocultures. (**A**) MatrisomeDB classifications of proteins either up-regulated or down-regulated in MDA-MB-231 Jag1WT and MEF coculture spheroids compared to Jag1KO cocultures. *n* = 4 biological replicates. (**B**) Proteins differentially expressed by either MDA-MB-231 cells or MEFs in MEF + Jag1WT cocultures compared to MEF + Jag1KO cocultures. (**C**) Cross sections of MDA-MB-231 (magenta) and MEF (orange) coculture spheroids stained with the CNA35 collagen probe (green) and DAPI (blue). Scale bars, 200 μm. Western blots and dot blots of indicated proteins in coculture spheroids of MDA-MB-231 cells and (**D**) MEFs or (**E**) human TIFs. Quantifications of four independent experiments are shown to the right. Data are presented as the means ± SEM. *P* values are calculated by a two-tailed unpaired *t* test. *P < 0.05, **P < 0.01, ***P < 0.001, and ****P < 0.0001.

As the Jag1-low tumors in the scRNA-seq data contained some cancer cells expressing high levels of Jagged1, we cannot rule out that interactions with Jagged1-expressing cancer cells may have still activated some of the Jag1-low fibroblasts. To clarify the effect of Jagged1 on fibroblasts and the ECM, we cocultured our Jag1WT or Jag1KO cells with mouse embryonic fibroblasts (MEFs) as spheroids in a U-bottom well 3D culture system without any externally added ECM. A mass spectrometry analysis on matrisome-enriched protein samples from these spheroids revealed a substantial up-regulation of collagen production in Jag1WT cocultures (Fig. 4A). The use of mouse fibroblasts and human cancer cells allowed us to perform a species-specific analysis on the proteomics results, showing cell type–specific changes in protein expression (Fig. 4B). Up-regulation of collagen production occurred both in cancer cells and in fibroblasts. However, when we stained spheroids with the fluorescent pan-collagen probe CNA35 [collagen-binding adhesion protein tagged with EGFP (enhanced green fluorescent protein)] (*31*), collagen mostly accumulated in the areas where fibroblasts resided in the spheroids (Fig. 4C). Another interesting observation was an apparent dysregulation of the TGFβ pathway, as several proteins related to the pathway exhibited Jagged1-dependent expression (Fig. 4B). Previously, we had seen a Jagged1-dependent up-regulation of a TGFβ gene signature both in cancer cells and in fibroblasts in the scRNA-seq data (Figs. 2C and 3E). Western blot analysis of spheroid samples showed increased phosphorylation of both SMAD2 and SMAD3, indicating increased TGFβ activity in the Jag1WT + MEF cocultures (Fig. 4D). Collagen production increased by almost 50% in the presence of Jagged1, whereas there was no difference in the production of FN. The differences in TGFβ activity and collagen production were even more notable when cancer cells were cocultured with human telomerase-immortalized fibroblasts (TIFs) as collagen production was increased by 70% and phosphorylation of SMAD2/3 by 40% in the presence of Jagged1, while FN appeared to be down-regulated in Jag1WT + TIF cocultures (Fig. 4E). The myCAF marker αSMA was also up-regulated by Jagged1 in both fibroblasts. Together, these results suggest that Jagged1 expressed by cancer cells induces collagen production and myCAF activation via increased TGFβ activity.

**Fig. 5.**
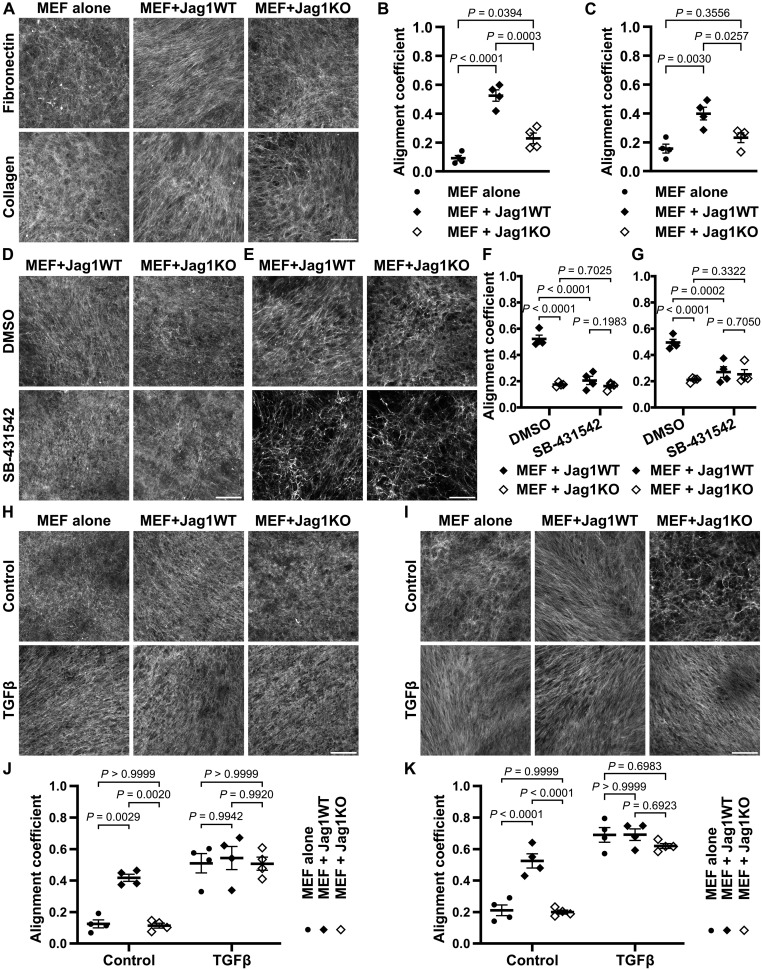
Jagged1 directs extracellular matrix fiber alignment via TGFβ signaling. (**A**) Representative images of CDMs from MEF monocultures or cocultures with MDA-MB-231 Jag1WT or Jag1KO cells and alignment coefficients of (**B**) FN and (**C**) collagen fibers. *P* values are calculated by a one-way analysis of variance (ANOVA) with Tukey’s multiple comparisons test. Representative images and fiber alignment coefficients of (**D** and **F**) FN and (**E** and **G**) collagen of CDMs from DMSO (dimethyl sulfoxide) or SB-431542 TGFβ inhibitor–treated Jag1WT or Jag1KO cocultures with MEF cells. *P* values are calculated by a two-way ANOVA. Representative images and fiber alignment coefficients of (**H** and **J**) FN and (**I** and **K**) collagen of CDMs from control or recombinant TGFβ1-treated MEF monocultures or MEF cocultures with Jag1WT or Jag1KO cells. *P* values are calculated by a two-way ANOVA with Tukey’s multiple comparisons test. Data are presented as the means ± SEM of four independent experiments. Scale bars, 100 μm.

**Fig. 6.**
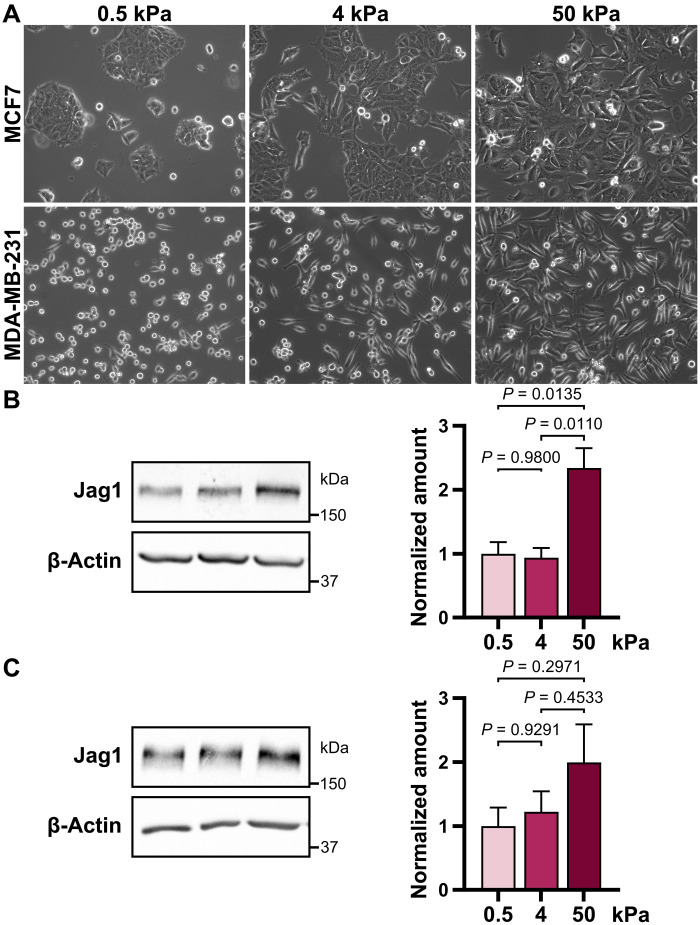
Matrix stiffness promotes Jagged1 protein expression. (**A**) Representative images of MCF7 and MDA-MB-231 WT cells grown on increasing substrate stiffness. Western blot analysis of Jag1 levels in (**B**) MCF7 and (**C**) MDA-MB-231 cells grown on increasing substrate stiffness. Quantifications of three independent experiments are shown to the right. Data are presented as the means ± SEM. *P* values are calculated by a one-way ANOVA with Tukey’s multiple comparisons test.

Along with the increased deposition of the ECM, especially fibrillar collagens, the increased cross-linking and stiffness of the ECM lead to the formation of a fibrotic, tumor-promoting microenvironment. The ECM surrounding the tumor is often anisotropically organized, and the densely packed and highly aligned ECM fibers create tracks for efficient cancer cell migration, thus promoting metastasis (*32*, *33*). As we have already established a significant increase in collagen deposition and altered expression of several matrix remodeling factors upon Jagged1 stimulation, we sought to evaluate whether Jagged1 is involved in regulating ECM fiber organization. When fibroblasts were cocultured with Jagged1-expressing cancer cells, the FN and collagen fibers of cell-derived matrices (CDMs) were highly aligned compared to fibroblast monocultures or cocultures with Jag1KO cells (Fig. 5, A to C, and fig. S6, A to C). Intriguingly, recombinant Jagged1 coating on the coverslips was able to induce an increase in fibroblast-produced CDM fiber alignment, whereas Notch ligands Dll1 and Dll4 lacked this ability (fig. S6, D and E). We hypothesized that this Jagged1-induced effect on ECM fiber alignment might be TGFβ-dependent, as Jagged1 was shown to increase TGFβ activity. Inhibition of TGFβ prevented the Jagged1-induced alignment of ECM fibers completely (Fig. 5, D to G), whereas recombinant TGFβ1 treatment rescued the Jag1KO phenotype (Fig. 5, H to K). Similar Jagged1-dependent ECM alignment was observed in another TNBC cell line, MDA-MB-436 (fig. S7, A to C). The induction of fiber alignment was dependent on Notch activation, as the gamma-secretase inhibitor PF-03084014 prevented the alignment of Jag1WT CDMs (fig. S7, D and E). Both the transcriptomics and proteomics analyses of Jag1WT samples revealed an up-regulation of matrix cross-linking LOXs, and inhibition of LOX also partly prevented the Jagged1-induced fiber alignment, although not as efficiently as Notch or TGFβ inhibition (fig. S8, A and B). There was no difference in the density of the FN matrix produced by Jag1WT or Jag1KO and MEF cocultures, indicating that the observed difference in FN fiber alignment is not affected by a differential FN concentration. In contrast, the lower density of the Jag1KO collagen matrix correlated with the lower overall collagen amount observed in Fig. 4D (fig. S8C). We observed a major reduction in the overall amount of ECM after TGFβ inhibition, whereas TGFβ1 treatment clearly induced ECM production. TGFβ modulation abrogated the difference seen in collagen density between Jag1WT and Jag1KO, further validating the notion that Jagged1 induces its effect on the ECM in a TGFβ-dependent manner (fig. S8, D and E). Expression of Jagged1 was increased upon culturing MDA-MB-231 as well as the ER+ MCF7 WT cells on increasing substrate stiffness, pointing toward a general feed-forward loop between ECM stiffness and Jagged1 expression, as both increased deposition and higher alignment of matrix fibers are indicative of increased tissue stiffness (Fig. 6). Together, Jagged1 is a central regulator of both ECM deposition and remodeling, inducing matrix fiber alignment through Notch and TGFβ signaling activity.

### Jagged1 promotes fibroblast activation and collagen deposition in vivo

**Fig. 7.**
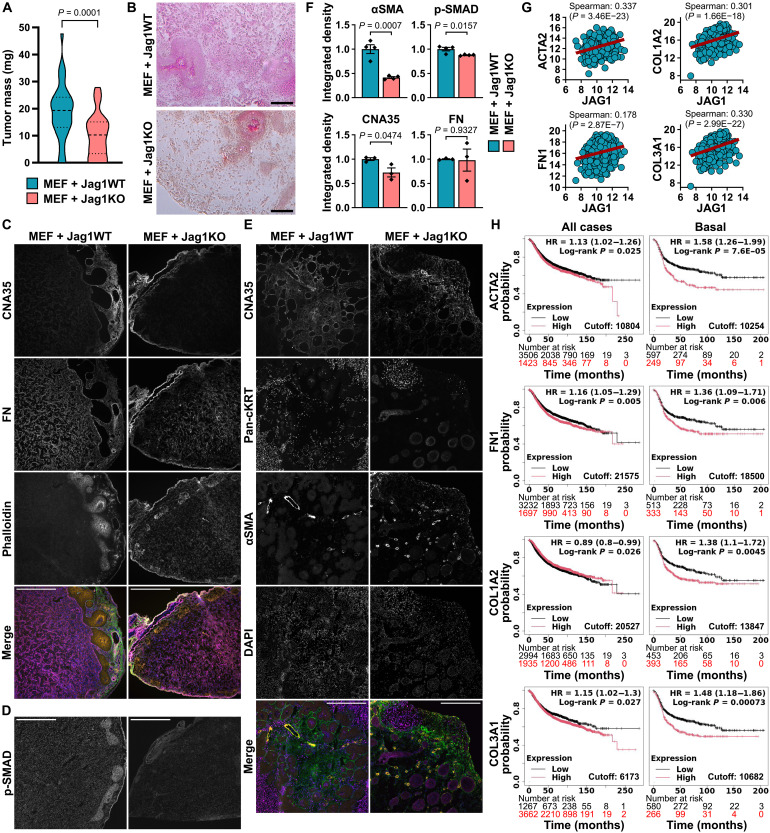
Jagged1 promotes collagen production and αSMA expression in vivo. (**A**) Tumor masses of MDA-MB-231 Jag1WT or Jag1KO cell cocultures with MEF cells in the chick CAM xenograft model. *P* values are calculated by a two-tailed unpaired *t* test. *n* = 37 tumors for Jag1WT and *n* = 40 for Jag1KO. (**B**) Hematoxylin and eosin–stained tissue sections of CAM tumors in (A). Scale bars, 100 μm. CAM tumor cryosections stained for (**C**) collagen (CNA35; green in merge), FN (magenta in merge), actin (phalloidin; orange in merge), and nuclei (DAPI; blue in merge); (**D**) phosphorylated SMAD2 (Ser^465/467^)/SMAD3 (Ser^423/425^) (p-SMAD); and (**E**) collagen (CNA35; green in merge), pan-cytokeratin (pan-cKRT; magenta in merge), αSMA (orange in merge), and nuclei (DAPI; blue in merge). Scale bars, 500 μm. (**F**) Quantifications of αSMA, p-SMAD, CNA35, and FN fluorescence integrated densities in CAM tumor cryosections. *n* = 4 tumors for αSMA and p-SMAD and *n* = 3 tumors for CNA35 and FN in each group. αSMA was quantified from glandular structures containing fibroblasts, and CNA35, FN, and p-SMAD were quantified from the whole-tumor area, excluding possible surrounding CAM. Data are presented as the means ± SEM. *P* values are calculated by a two-tailed unpaired *t* test. (**G**) mRNA expression level correlation with Jag1 mRNA expression in patients with breast cancer (TCGA, Cell 2015, *n* = 817) (*26*) and (**H**) relapse-free survival of patients with low (black) or high (red) expression of *ACTA2*, *FN1*, *COL1A2*, or *COL3A1* in all breast cancer cases and in basal breast cancer separately in the Kaplan-Meier Plotter database (*58*).

**Fig. 8.**
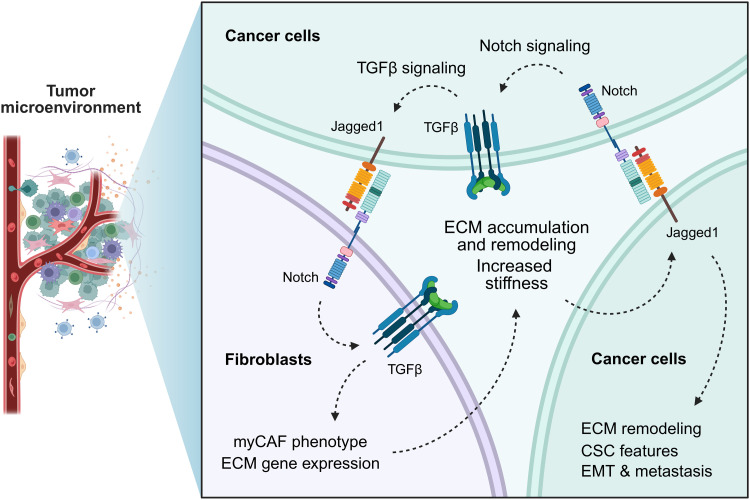
Jagged1-mediated cross-talk between TNBC cells and fibroblasts enhances myofibroblast activation, ECM accumulation, and alignment of ECM fibers via increased Notch activation and TGFβ activity. High Jagged1 expression promotes CSC-like features, EMT, and invasiveness of TNBC cells, thus enhancing tumor formation and metastasis. Jagged1 expressed by cancer cells activates Notch in the surrounding fibroblasts and increases TGFβ activity. Fibroblasts interacting with Jagged1-expressing cancer cells transdifferentiate into contractile myCAFs with increased deposition of ECM, especially collagen, and an increased capability of tumorigenic remodeling and aligning of the ECM fibers. In cancer cells, increased stiffness of the surrounding tissue and TGFβ activity induce further expression of Jagged1, leading to a tumor-promoting feed-forward loop. Jagged1 is a central modulator of the TNBC tissue structure, acting upstream of TGFβ. Created in BioRender. M. Parikainen (2025), https://biorender.com/o4se15z.

To determine whether Jagged1 regulates fibroblast behavior and ECM production in vivo, we cocultured MEFs with either Jag1WT or Jag1KO cells in the CAM model. Jag1WT tumors were significantly bigger (average mass of Jag1WT tumors was 18.8 mg, and that of Jag1KO tumors was 10.3 mg), and on the basis of hematoxylin and eosin staining, they showed more histological resemblance to invasive breast cancer than Jag1KO tumors while demonstrating stronger eosin staining, possibly pointing to increased collagen (Fig. 7, A and B). We next analyzed tumor sections for the expression of collagen, FN, αSMA, and the level of phosphorylated SMAD2/3 as a marker of TGFβ activity (Fig. 7, C to F). αSMA-positive cells appeared to reside in glandular structures within the tumor, surrounded by pan-cytokeratin–positive cancer cells of epithelial origin. Similar separation of fibroblast and cancer cell location was seen previously in coculture spheroids (Fig. 4C). Blood vessels also showed bright αSMA staining because of smooth muscle cells in the vessel walls (Fig. 7E and fig. S9A). However, the αSMA-positive glandular structures did not show any positivity for the smooth muscle cell marker desmin (fig. S9, B and C), suggesting that these glandular structures are separate from the CAM-derived vasculature and contain xenografted cells. We quantified αSMA expression in these glandular structures, and it was 2.4 times higher in Jag1WT tumors, indicating Jagged1-induced myCAF differentiation (Fig. 7F). There was also a 27% increase in the amount of collagen in the whole-tumor area, even when cells were grafted in collagen-containing Matrigel, while the level of phosphorylated SMAD2/3 was 12% higher in Jag1WT tumors. No differences were seen in FN deposition, in line with our previous results from MEF coculture spheroids (Fig. 4D). Both αSMA (*ACTA2*) and multiple different collagens showed a positive RNA expression level correlation with Jagged1 in human datasets of patients with breast cancer (*26*) and displayed a prognostic value, especially in basal breast cancer, similar to Jagged1, while the correlation between FN and Jagged1 was much weaker (Fig. 7, G and H, and fig. S10A). Jagged1 expression also correlated positively with many matrix-remodeling enzymes, components of the TGFβ pathway, and several integrins in the patient dataset (fig. S10) (*26*), as also seen in our RNA sequencing and proteomics data and the scRNA-seq data (*25*). Together, our data establish Jagged1 as an important modulator of tumor tissue structure upstream of TGFβ in TNBC (Fig. 8).

## DISCUSSION

The Notch signaling pathway is an attractive target for breast cancer treatment because of its roles in supporting CSCs, tumor heterogeneity, and chemoresistance (*34*–*36*). However, pan-Notch inhibition has failed clinically because of severe off-target effects, such as gastrointestinal toxicity, and the inherent complexity of the pathway, where different Notch receptors and ligands play distinct and sometimes opposing roles depending on the tumor context. Targeting specific Notch components requires a precise understanding of their context-dependent roles. Here, we show that the Notch ligand Jagged1 is highly expressed in TNBC, where it correlates with poor prognosis. This is in line with previous reports showing a higher expression of Jagged1 in aggressive breast cancer subtypes (*20*, *21*, *23*, *24*). We demonstrate how Jagged1 drives tumor growth and invasion by inducing CSC properties, EMT, and remodeling of the ECM in TNBC. Kumar *et al.* (*37*) showed a similar ligand-specific role for Dll1 in promoting luminal ER+ breast cancer but not TNBC. In contrast, Yamamoto and colleagues (*38*) described an expansion of CSCs via nuclear factor κB–dependent induction of Jagged1 that occurred only in basal-like breast cancer. These observations further strengthen the role of individual Notch ligands in distinct breast cancer subtypes.

Breast tissue goes through major alterations during the progression toward an invasive tumor. Increased deposition of fibrillar collagens and substantial remodeling of the ECM lead to increased tissue stiffness, driving tumor progression and metastasis (*3*–*5*). TGFβ signaling is a major driver of adverse tissue remodeling in aggressive cancer and fibrosis (*39*). Our data demonstrate that Jagged1 promotes collagen accumulation and ECM fiber alignment through cancer cell-fibroblast cross-talk upstream of TGFβ, thus modulating the tissue structure. We further demonstrate that higher stiffness increases Jagged1 expression. TGFβ has been shown to induce Jagged1 expression (*22*, *40*, *41*), pointing toward a vicious cycle where Jagged1 induces increased tissue stiffness and TGFβ activity, which then further promote Jagged1 expression and tumor progression. Jagged1 can also cross-talk with the TGFβ pathway independently of Notch receptors by forming a transcriptional complex containing J1ICD, SMAD3, DDX17, and TGIF2 (*42*). While we consistently observe a Jagged1-induced, TGFβ-dependent accumulation of collagen across our in vitro and in vivo experiments, the amount of FN does not follow this pattern and is increased in Jag1KO cocultures with TIF cells despite the reduced TGFβ activity. Although TGFβ regulates the synthesis of both collagen and FN in a similar manner, the accumulation of FN in the matrix also depends on the presence of appropriate cell-surface adhesion receptors. Unlike collagens, FN is highly sensitive to degradation by various serine proteases and matrix metalloproteinases. While we do observe a positive correlation between Jagged1 and FN at the RNA level in patient data, we do also see Jagged1-dependent differences in the expression of multiple matrix-degrading enzymes, such as MMP-7 and MMP-9. Hence, differential degradation may explain the discrepancies in protein levels we observe. Notably, the alignment of FN fibers does not appear to depend on its concentration, as Jagged1 induced fiber alignment in both MEF and TIF cell cocultures. The exact mechanisms by which Jagged1 cross-talks with TGFβ and different types of fibroblasts in TNBC should be further elucidated.

CAFs are the major regulators of ECM remodeling in tumors. CAFs display substantial phenotypic and functional heterogeneity, with their highly variable context-dependent subsets and cellular origins leading to a lack of definitive markers to distinguish CAFs from all other cell types of the TME, such as pericytes and smooth muscle cells (*6*–*9*). Matrix-producing myCAFs, typically found in close proximity to cancer cells, express high αSMA and other TGFβ-responsive genes (*43*). Our data indicate that Jagged1-Notch signaling between cancer cells and fibroblasts drives myCAF activation, characterized by increased αSMA expression, TGFβ signaling, and matrix production. In our scRNA-seq data analysis, we did see some fibroblast clusters expressing markers of pericytes and adipocytes, suggesting that the data might contain falsely annotated cells or that the fibroblasts originated from these cell types and retain some marker gene expression. As, especially, pericytes can express similar contractility markers as myofibroblasts and have a high Notch gene signature, we reanalyzed the fibroblast scRNA-seq data without the pericyte marker–positive clusters. Even after the reanalysis, we could see a Jagged1-dependent increase in myCAF, Notch, and TGFβ marker gene expression, further validating our conclusion of Jagged1-Notch–induced myCAF activation. Previous data show that αSMA is a direct Notch target gene (*44*), and other studies confirm Notch-dependent αSMA induction through cell-cell contact (*13*, *16*, *45*–*47*). Pelon and colleagues (*15*) identified four subpopulations of breast CAFs, with the subset CAF-S4 displaying a significant up-regulation of Notch receptors 1 to 3 and demonstrating matrix remodeling and metastasis-promoting functionality. The same group later identified five subpopulations of myCAFs, with TGFβ-myCAFs distinguished by a high TGFβ gene signature (*30*). Direct contact with cancer cells, but not cancer cell–conditioned medium, induced TGFβ-myCAF differentiation, while *NOTCH3* and *HES4* were reported among the marker genes of this fibroblast subpopulation, suggesting Notch dependency. Our data show that Jagged1 induces differentiation of fibroblasts with a TGFβ signature and a highly similar set of marker genes as TGFβ-myCAFs, including Notch target genes and *LAMP5*. A higher abundance of TGFβ-myCAFs predicted progression of ductal carcinoma in situ into invasive breast cancer (*48*), making them an attractive therapy target.

Previous data support the reversibility of the myofibroblastic activation state of CAFs, making CAF targeting an exciting treatment opportunity (*49*–*51*). Targeting the stromal cells by TGFβ blockade or direct targeting of CAF markers has been shown to inhibit tumor growth and trigger antitumor immune responses (*52*–*54*). Our results indicate that Jagged1 is important in mediating myCAF activation, TGFβ signaling, and the development of tumor fibrosis in TNBC, suggesting that therapeutically targeting Jagged1 could be a promising approach to control the CAF phenotype and adverse TME remodeling. Notably, targeting Jagged1 may be beneficial only against the more aggressive breast cancer subtypes, as we have shown that it is only in these subtypes that the Jagged1 expression level has a prognostic value for patient survival. In accordance with our data, a TGFβ-dependent formation of a reactive stroma through Jagged1 overexpression in cancer cells was reported in a Pten null mouse model of prostate cancer (*55*), potentially extending the scope of disease contexts that might benefit from Jagged1-targeted therapy. Possible challenges and unanswered questions with this approach include whether an already formed ECM structure is reversible and whether excessive reversal of the fibrotic ECM state will lead to tumor collapse, hampering drug delivery and immune infiltration while releasing matrix-embedded growth factors and cytokines. Future studies will need to focus on a more comprehensive analysis of Jagged1-mediated effects on other cell types within the TME besides fibroblasts, such as immune cells and adipocytes. Because of the pleiotropic effects and context dependency of Notch signaling in the TME, the correct target and timing of Notch-based therapeutics must also be carefully planned.

## MATERIALS AND METHODS

### Cell culture and treatments

Human breast cancer cell lines MDA-MB-231, MDA-MB-436, SK-BR-3, mouse fibroblast cell line MEF, and human fibroblast cell line TIF (a gift from J. Ivaska, University of Turku, Turku, Finland) were grown in high-glucose Dulbecco’s modified Eagle’s medium (DMEM), supplemented with 10% fetal bovine serum, 2 mM glutamine, penicillin (100 units/ml), and streptomycin (100 μg/ml). The human breast cancer cell line MDA-MB-361 was supplemented with 20% fetal bovine serum. Human breast cancer cell lines BT474, HCC1954, and T47D were grown in RPMI instead of DMEM, with the same supplements. The human mammary epithelial cell line MCF10A was grown in DMEM/F12, supplemented with 5% horse serum, 2 mM glutamine, penicillin (100 units/ml), streptomycin (100 μg/ml), epidermal growth factor (20 ng/ml), hydrocortisone (0.5 μg/ml), cholera toxin (100 ng/ml), and insulin (10 μg/ml). All cells were maintained at 37°C with 5% CO_2_ and regularly screened for mycoplasma infections. TGFβ receptor kinase inhibitor SB-431542 (10 μM; MedChemExpress), 10 μM gamma-secretase inhibitor PF-03084014 (MedChemExpress), 500 μM LOX inhibitor β-aminopropionitrile (MedChemExpress), or recombinant human TGFβ1 (10 ng/ml; PeproTech) were added to the medium where stated. For siRNA-mediated Jagged1 silencing, a cocktail of two siRNAs with the following sequences was used at a final concentration of 25 nM: 5′-GGG AUU UGG UUA AUG GUU A(dTdT)-3′ and 5′-GAA CCA CAG CAA CGA TCA CAA(dTdT)-3′. The nontargeting control siRNA sequence was 5′-CCU ACA UCC CGA UCG AUG AUG(dTdT)-3′. siRNAs were transfected with the Lipofectamine RNAiMAX transfection reagent (Invitrogen) according to the manufacturer’s instructions.

### Generation of Jagged1 knockout and Jagged1 rescue cells

MDA-MB-231 WT cells have endogenous expression of Jagged1. To create a Jagged1-negative cell line, single guide RNA 5′-TATCAGTCCCGCGTCACGGC-3′, targeting Jagged1 exon 2, was cloned into pSPCas9(BB)-2A-GFP (PX458) (Addgene, no. 48138) (*56*), and the construct was transfected into Jag1WT cells. Transfected cells were sorted using flow cytometry as single cells on a 96-well plate on the basis of GFP expression, allowed to form colonies, and then checked for successful Jagged1 knockout using DNA sequencing and Western blotting. Fifteen single-cell clonal Jag1KO populations were mixed in equal ratios to create the final Jag1KO cell line. Jag1KO cells were then transfected either with a pcDNA3.1 vector carrying Jagged1 cDNA to generate the Jag1KO + rescue cell line or with an empty pcDNA3.1 vector to create the negative control cell line Jag1KO + ctrl. Jag1KO + rescue and Jag1KO + ctrl cells were grown in the presence of G418 (500 μg/ml) selection.

### CAM model

For monocultures, 1 × 10^6^ MDA-MB-231 cells were transplanted onto the CAM of fertilized chicken eggs on embryonic day 8 in 20 μl of Matrigel (4 mg/ml; Corning, no. 356231) and allowed to form tumors for 5 days. Simple randomization was used to allocate eggs into different sample groups. Afterward, tumors were excised from the CAM and weighed. For cocultures, 1 × 10^6^ MDA-MB-231 cells and 1 × 10^6^ MEF cells were used. Optimal cutting temperature compound–embedded CAM tumors were snap-frozen with liquid nitrogen and stored in −80°C until further processing.

### Zebrafish invasion assay

The zebrafish used in experiments were bred and handled in the Zebrafish Core of Turku Bioscience Centre under license MMM/465/712-93 (Ministry of Agriculture and Forestry in Finland). The xenotransplantation of zebrafish embryos was performed as described in (*57*). MDA-MB-231 cells were stained with 10 μM CellTracker Green CMFDA dye (Thermo Fisher Scientific) before xenotransplantation. A cell suspension (2.3 nl) in phosphate-buffered saline (PBS), containing 200 cells, was microinjected into the duct of Cuvier/common cardinal vein of 2-days-postfertilization zebrafish pigmentless casper strain (roy−/−; mitfa−/−) embryos. Simple randomization was used to allocate embryos into different sample groups. The following day, embryos were imaged with a Nikon Eclipse Ti2-E microscope, with a Nikon Plan-Apochromat 2×/0.06 objective, and invaded cells were analyzed with ImageJ/FIJI software, as described in (*57*). The number of invaded cells in zebrafish tails was counted manually by adjusting the brightness and contrast of the original images to distinguish individual cells. The image analysis was performed blinded.

### Kaplan-Meier survival plots

Relapse-free survival of patients with breast cancer was assessed in the Kaplan-Meier Plotter database for breast cancer (*58*). The JetSet optimal probe set was used for each gene, and patients were split by autoselect best cutoff. StGallen molecular breast cancer subtypes were used to visualize subtype-specific survival, and ER array status was used to split patients into ER+ and ER− subgroups.

### Single-cell data processing

The scRNA-seq data generated by Bassez *et al.* (*25*) were obtained from https://lambrechtslab.sites.vib.be/en/single-cell. The available filtered count matrices and cell annotations were used as a starting point and reprocessed using the R package Seurat version 5 (*59*). The code used in processing the data is provided with this paper (data file S1). Briefly, treatment-naïve cohort 1 samples were retrieved, low-quality cells were filtered on the basis of the quality control distribution for each cell type, and data were log normalized and scaled with default parameters. An expression value of 0.5 for JAG1 was used to split TNBC cells into Jag1-high and Jag1-low populations. For fibroblasts, a clustering resolution of 0.5 was used, and small subclusters of endothelial and T cells were removed on the basis of cell type marker expression. Patients with at least 50 cancer cells in the tumor sample were retained for analysis of fibroblasts to assess tumor Jag1 status reliably. The GSEAs were conducted with Metascape (*60*) using the cell type–specific transcriptomes in the dataset as background genes.

### 3D Matrigel cultures

Cell culture plates were first coated with a thin layer of Matrigel (4 mg/ml; Corning, no. 356231). For 96-well plates, 5 × 10^3^ cells per well were seeded in 60 μl of Matrigel (2 mg/ml). For six-well plates, 5 × 10^4^ cells per well were seeded in 600 μl of Matrigel (2 mg/ml). siRNA transfections were performed 7 hours before cell seeding. Matrigel was allowed to solidify overnight, after which the prewarmed cell culture medium was added on top of the 3D cultures. Spheroids were allowed to grow for 7 days. The fresh medium was changed once on day 4. The gamma-secretase inhibitor PF-03084014–containing medium was changed every 24 hours. Spheroids were extracted from Matrigel for further analysis with 5 mM EDTA in PBS. Briefly, 3D cultures were washed three times with ice-cold PBS before adding ice-cold EDTA/PBS and transferring the cultures to Falcon tubes on ice. Matrigel was allowed to dissolve for 15 min before collecting the spheroids by centrifugation.

### RNA sequencing and transcriptomics data processing

RNA was isolated using the NucleoSpin RNA kit (MACHEREY-NAGEL, no. 740955). The sequencing library was prepared using the Illumina TruSeq Stranded mRNA HT Sample Preparation kit, and sequencing was performed using an Illumina NovaSeq 6000 SP with a 2 × 50–base pair read length at the Finnish Functional Genomics Centre in Turku, Finland. Reads were quality checked and postprocessed at the Medical Bioinformatics Centre, Turku, Finland. Briefly, reads were aligned against the human reference genome (hg38), and uniquely mapped reads were used to generate gene-wise read counts. TMM (trimmed mean of *M* values) normalization from the Bioconductor package edgeR was used to normalize the filtered gene counts between the samples. Normalized counts as CPM (counts per million) values were used as input in further analysis. Genes with more than 1 CPM expression in at least 50% of the replicates were retained in the analysis. The raw count data were transformed to CPM offset by 1, log_2_ transformed, and then further analyzed using the Bioconductor package ROTS to acquire fold change and false discovery rate (fdr) values. The GSEA was conducted using Metascape (*60*).

### Gene expression in datasets of patients with breast cancer

Data of patients with breast cancer were accessed and downloaded through the cBioPortal service (*61*–*63*). Jagged1 mRNA coexpression *z*-scores were evaluated relative to RNA sequencing diploid samples in a breast invasive carcinoma dataset [The Cancer Genome Atlas (TCGA), Cell 2015] (*26*), with *n* = 817 consisting of 808 female and 9 male patients of varied ethnicity and race. Simple linear regression and Spearman’s rank correlation were used for modeling the correlation between mRNA expression levels, and *P* values were calculated by a two-tailed unpaired *t* test.

### Proteomics of spheroid samples

Spheroids were made using no. 24-35 micromolds from 3D Petri Dish, MicroTissues, according to the manufacturer’s instructions, with 1.4 × 10^5^ MEFs and 1.4 × 10^5^ of either Jag1WT or Jag1KO cells mixed and added into one mold. The medium was supplemented with ascorbic acid (50 μg/ml) and changed daily. After 15 days of culture, spheroids were collected, and samples were processed and analyzed by liquid chromatography–mass spectrometry, as described in (*64*). DIA files were processed with Spectronaut (version 16.3, Biognosys), as described in (*64*), using human UniProt-reviewed sequences, along with their isoforms (release 2022_01), as a reference. A maximum of five variable modifications per peptide were allowed. A minimum peptide length of seven amino acids with a maximum peptide length of 52 amino acids, containing a maximum of two missed cleavages, was allowed. The statistical analysis was performed using Perseus (version 1.6.14.0) on the basis of a customized pivot report containing the MS2 quantification data from Spectronaut. The MS2 quantities were log_2_ transformed and normalized with width adjustment. Welch’s *T* test with Benjamini-Hochberg fdr was used. ECM proteins were identified using the MatrisomeDB database (https://matrisomedb.org/) (*65*).

### Immunofluorescence staining of spheroid samples

For staining of coculture spheroids, MDA-MB-231 and MEF cells were stained with 10 μM CellTracker Orange CMRA and CellTracker Deep Red dyes (Thermo Fisher Scientific), respectively, before mixing cells together. Spheroids were grown in the same way as mentioned previously in the “Proteomics of spheroid samples” section. After 15 days of culture, spheroids were collected by centrifugation, washed with PBS, and then fixed and permeabilized in PBS containing 4% paraformaldehyde and 1% Triton X-100 for 3 hours at +4°C. After fixation, spheroids were washed in PBST (0.1% Triton X-100 in PBS) for 15 min and then incubated in blocking buffer [3% bovine serum albumin (BSA) in PBST] for 3 hours at +4°C. Spheroids were washed for 15 min in PBST and then incubated in CNA35 [20 ng/μl; pET28a-EGFP-CNA35 was a gift from M. Merkx; Addgene, plasmid no. 61603 (*31*)] and 600 nM 4′,6-diamidino-2-phenylindole (DAPI) in PBST overnight at +4°C with gentle shaking. Spheroids were washed three times in PBST and mounted on microscopy slides in glycerol. A Zeiss LSM880 Axio Observer.Z1 confocal microscope and ZEN 2.3 SP1 black edition software were used for imaging. The objective used was a Zeiss Plan-Apochromat 20×/0.8.

### Western blotting

Spheroids were lysed on ice with Laemmli SDS sample buffer containing 3% β-mercaptoethanol, and protein lysates were denatured by boiling. Proteins were separated by SDS–polyacrylamide gel electrophoresis and transferred to a nitrocellulose membrane using a wet transfer apparatus, followed by blocking with 5% nonfat dry milk in tris-buffered saline (TBS). Membranes were incubated with primary antibodies diluted in 3% BSA/0.02% NaN_3_ overnight at +4°C, followed by incubation with a secondary antibody, coupled to horseradish peroxidase, for 1 hour at room temperature (RT). The following antibodies and dilutions were used: 1:1000 Jagged1 (Cell Signaling Technology, cat. no. 2620, RRID: AB_10693295), 1:1000 phospho-Smad2 (Ser^465/467^)/Smad3 (Ser^423/425^) (Cell Signaling Technology, cat. no. 8828, RRID: AB_2631089), 1:1000 Smad2/3 (Santa Cruz Biotechnology, cat. no. sc-133098, RRID: AB_2193048), 1:1000 αSMA (Cell Signaling Technology, cat. no. 19245, RRID: AB_2734735), 1:2500 FN (BD Biosciences, cat. no. 610077, RRID: AB_2105706), 1:1000 ERα (Santa Cruz Biotechnology, cat. no. sc-8002, RRID: AB_627558), 1:1000 β-tubulin (Cell Signaling Technology, cat. no. 86298, RRID: AB_2715541), 1:200,000 β-actin (Sigma-Aldrich, cat. no. A1978, RRID: AB_4766922), and 1:5000 HSC70 (Enzo Life Sciences, cat. no. ADI-SPA-815, RRID: AB_10617277). Proteins were detected with SuperSignal West Pico PLUS Chemiluminescent Substrate (Thermo Fisher Scientific) using the iBright FL1500 Imaging System (Invitrogen). The results were normalized against housekeeping protein expression and by using the sum-of-all-data-points method. For collagen visualization, the dot blot technique was used. Cell lysates were directly spotted on a nitrocellulose membrane, allowed to dry, rinsed with MQ-H_2_O, and stained for total protein using the Revert 700 Total Protein Stain Kit (Li-COR) according to the manufacturer’s instructions. After destaining, nonspecific binding was blocked with 5% nonfat dry milk in TBS, and the membrane was incubated with CNA35 (20 ng/μl) in PBS for 1 hour at RT. The membrane was washed with TBS, and the GFP signal was imaged using the iBright FL1500 Imaging System (Invitrogen). ImageJ/FIJI software was used to quantify images. Unprocessed Western blots are provided in the Supplementary Materials.

### Cell-derived matrices

CDMs were produced as previously described in (*66*). Briefly, 6 × 10^5^ fibroblast cells for monocultures or 2 × 10^5^ MDA-MB-231 cells and 4 × 10^5^ fibroblast cells for cocultures were plated on gelatin-coated coverslips and allowed to form a confluent monolayer overnight. The following day, treatment with ascorbic acid (50 μg/ml) was started and continued for 12 or 15 days for TIF or MEF cocultures, respectively. The ascorbic acid–containing medium was changed daily. After cell denudation, CDMs were fixed and stained as described in (*66*). For FN staining, a mouse anti-FN antibody (2.5 μg/ml; BD Biosciences, cat. no. 610077, RRID: AB_2105706) was used, followed by an anti-mouse Alexa Fluor 555–conjugated secondary antibody (4 μg/ml; Molecular Probes, cat. no. A-21424, RRID: AB_141780). Collagen was visualized using CNA35 (20 ng/μl), incubated together with the secondary antibody. CDMs were imaged using a Zeiss LSM880 Axio Observer.Z1 confocal microscope using ZEN 2.3 SP1 black edition software. The objective used was a Zeiss Plan-Apochromat 20×/0.8. One *z* slice from the middle of the sample was captured, and five images per sample at randomized positions were acquired for analysis. Fiber alignment coefficients were analyzed with CurveAlign version 4.0 beta software (*67*, *68*). Fibers in the image boundary were excluded from analysis, and the third finest scale was used.

### Recombinant Notch ligand coating

Gelatin-coated coverslips were incubated with Recombinant Protein G (50 μg/ml; Thermo Fisher Scientific) in PBS at RT overnight. Coverslips were washed three times with PBS, blocked with 1% BSA/PBS for 1 hour at RT, and washed three times with PBS. Coverslips were then incubated with 10 nM recombinant human Fc-linked Notch ligands (R&D Systems; Jag1, no. 1277-JG; Dll1, no. 10184-DL; Dll4, no. 10185-D4) or 10 nM recombinant human Fc-linked IgG1 (immunoglobulin G1; R&D Systems, no. 110-HG) in 0.1% BSA/PBS overnight at +4°C. Coverslips were washed three times with PBS before plating cells for CDM production.

### CAM cryosection staining

Snap-frozen CAM tumors in optimal cutting temperature compound were cut into 8-μm cryosections for immunofluorescence staining and 6-μm cryosections for hematoxylin and eosin staining. For immunofluorescence staining, cryosections were fixed and permeabilized with 4% paraformaldehyde/1% Triton X-100 in PBS for 10 min at RT, washed with PBS, and incubated in blocking buffer (10% horse serum in PBS) for 30 min at RT. Primary antibodies against FN (BD Biosciences, cat. no. 610077, RRID: AB_2105706), αSMA (Cell Signaling Technology, cat. no. 19245, RRID: AB_2734735), pan-cytokeratin (Sigma-Aldrich, cat. no. C2562, RRID: AB_476839), phospho-Smad2 (Ser^465/467^)/Smad3 (Ser^423/425^) (Cell Signaling Technology, cat. no. 8828, RRID: AB_2631089), and desmin (Bio SB, cat. no. BSB 5460, RRID: AB_3716523) were diluted 1:100 in blocking buffer and incubated on cryosections overnight at +4°C. The following day, cryosections were washed three times with PBST and incubated with secondary antibodies (4 μg/ml; anti-mouse Alexa Fluor 555, Molecular Probes, cat. no. A-21424, RRID: AB_141780; anti-rabbit Alexa Fluor 647, Molecular Probes, cat. no. A-31573, RRID: AB_2536183), 165 nM Alexa Fluor 633 Phalloidin (Invitrogen, no. A22284), CNA35 (20 ng/μl), and 600 nM DAPI in blocking buffer for 1 hour at RT. Cryosections were washed three times with PBS, rinsed once with MQ-H_2_O, and mounted with Mowiol/DABCO on microscopy slides. A Zeiss LSM880 Axio Observer.Z1 confocal microscope and ZEN 2.3 SP1 Black Edition software were used for imaging, with a Zeiss EC Plan-Neofluar 10×/0.30 objective. Integrated signal densities were quantified using ImageJ/FIJI software.

### Quantitative PCR

RNA was isolated using the NucleoSpin RNA kit (no. 740955, MACHEREY-NAGEL), and cDNA was prepared from 1 μg of RNA using the SensiFAST cDNA Synthesis kit (Bioline). The reaction mixtures for quantitative PCR were prepared with 5xHOT FIREPol EvaGreen qPCR Mix Plus with ROX (Solis BioDyne) at a 10-μl final reaction volume, containing 4 μl of cDNA and primers at a 250 nM final concentration. Primer sequences are provided in table S8. Cycle threshold (CT) values were normalized to UBC CT values, and fold changes were calculated with the 2−ΔΔCt formula

### Protein expression on increasing substrate stiffness

MDA-MB-231 or MCF7 WT cells were plated on Matrigen Softwell Petrisoft 35-mm Dish Collagen (Cell Guidance Systems) dishes with 0.5-, 4-, or 50-kPa stiffness values for 72 hours before cell lysis with Laemmli SDS sample buffer containing 3% β-mercaptoethanol. Lysates were analyzed by Western blotting, as described above.

### Statistical analysis

Statistical analyses and illustrations were prepared using GraphPad Prism 10.3.1 or R/R-Studio (version 4.4.2). Experiments were independently repeated a minimum of three times. Detailed information about exact *n* values, displayed data and error bars, and statistical tests are reported in the figure legends. The normality of the data was tested using the Shapiro-Wilk test before the use of any parametric tests. The ROUT method (*Q* = 0.5%) was used to detect and remove two outliers from Jag1KO zebrafish invasion data in Fig. 1F. Statistical source data are provided in data file S2.

## Supplementary Materials

### This PDF file includes:

Figs. S1 to S10 Legends for tables S1 to S7 Table S8 Legends for data files S1 and S2 Unprocessed Western blots
