# Supplementary material for: Jagged1 regulates extracellular matrix deposition and remodeling in triple-negative breast cancer
Source: Sci Adv. 2026 Mar 18;12(12):eaea9562. doi: 10.1126/sciadv.aea9562 (PMC12998526; doi:10.1126/sciadv.aea9562)
Supplement: Supplementary file 1 — Figs. S1 to S10 Legends for tables S1 to S7 Table S8 Legends for data files S1 and S2 Unprocessed Western blots [file sciadv.aea9562_sm.pdf]

Science Advances
AAAS

Supplementary Materials for

Jagged1 regulates extracellular matrix deposition and remodeling in triple-negative breast cancer

Marjaana Parikainen *et al.*

Corresponding author: Cecilia M. Sahlgren, cecilia.sahlgren@abo.fi

Sci. Adv. **12**, eaea9562 (2026)
DOI: 10.1126/sciadv.aea9562

The PDF file includes:

Figs. S1 to S10
Legends for tables S1 to S7
Table S8
Legends for data files S1 and S2
Unprocessed Western blots

Other Supplementary Material for this manuscript includes the following:

Tables S1 to S7
Data files S1 and S2

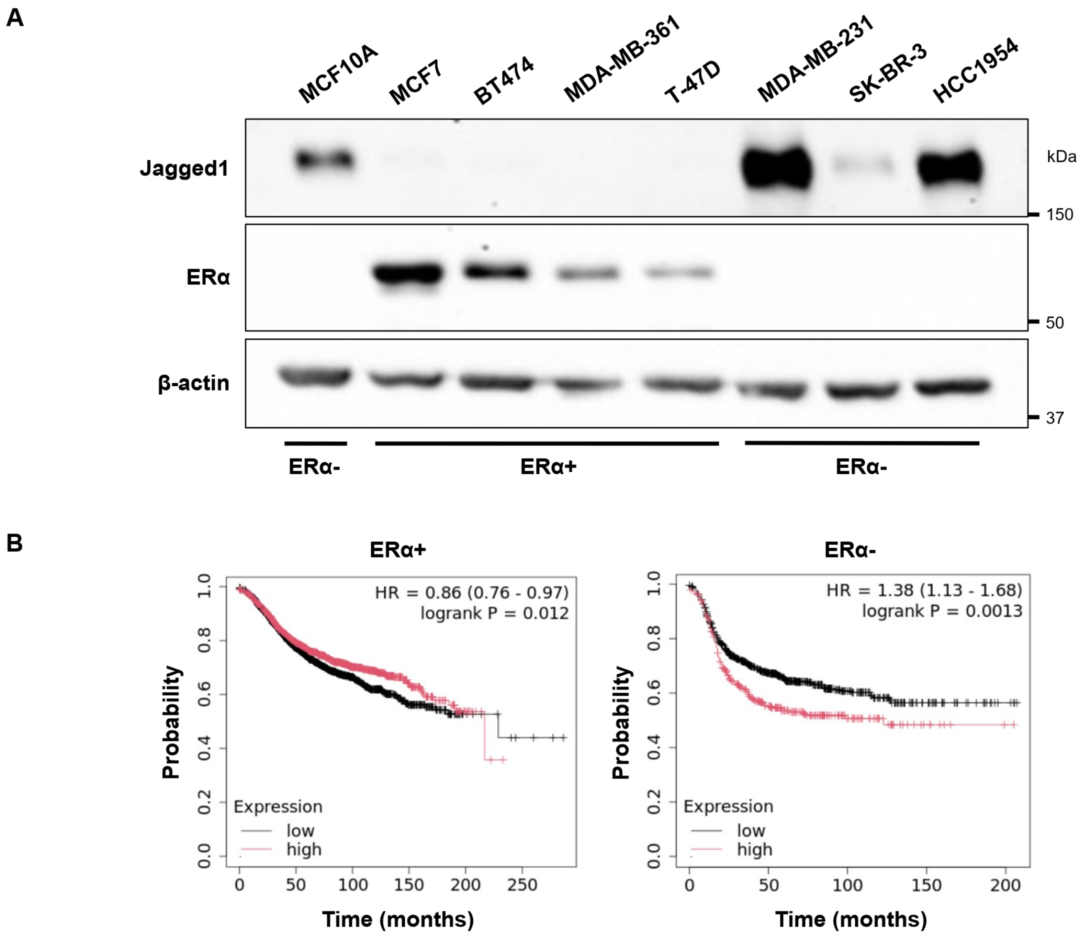

Fig. S1. High Jagged1 expression is associated with estrogen receptor (ERα) negative breast cancer. (**A**) Western blots showing Jagged1 and ERα expression levels in a panel of breast cancer cell lines and in the non-tumorigenic, ERα negative breast epithelial cell line MCF10A. (**B**) Relapse-free survival of patients with ERα+ or ERα- breast cancer and either low (black) or high (red) expression of Jagged1.

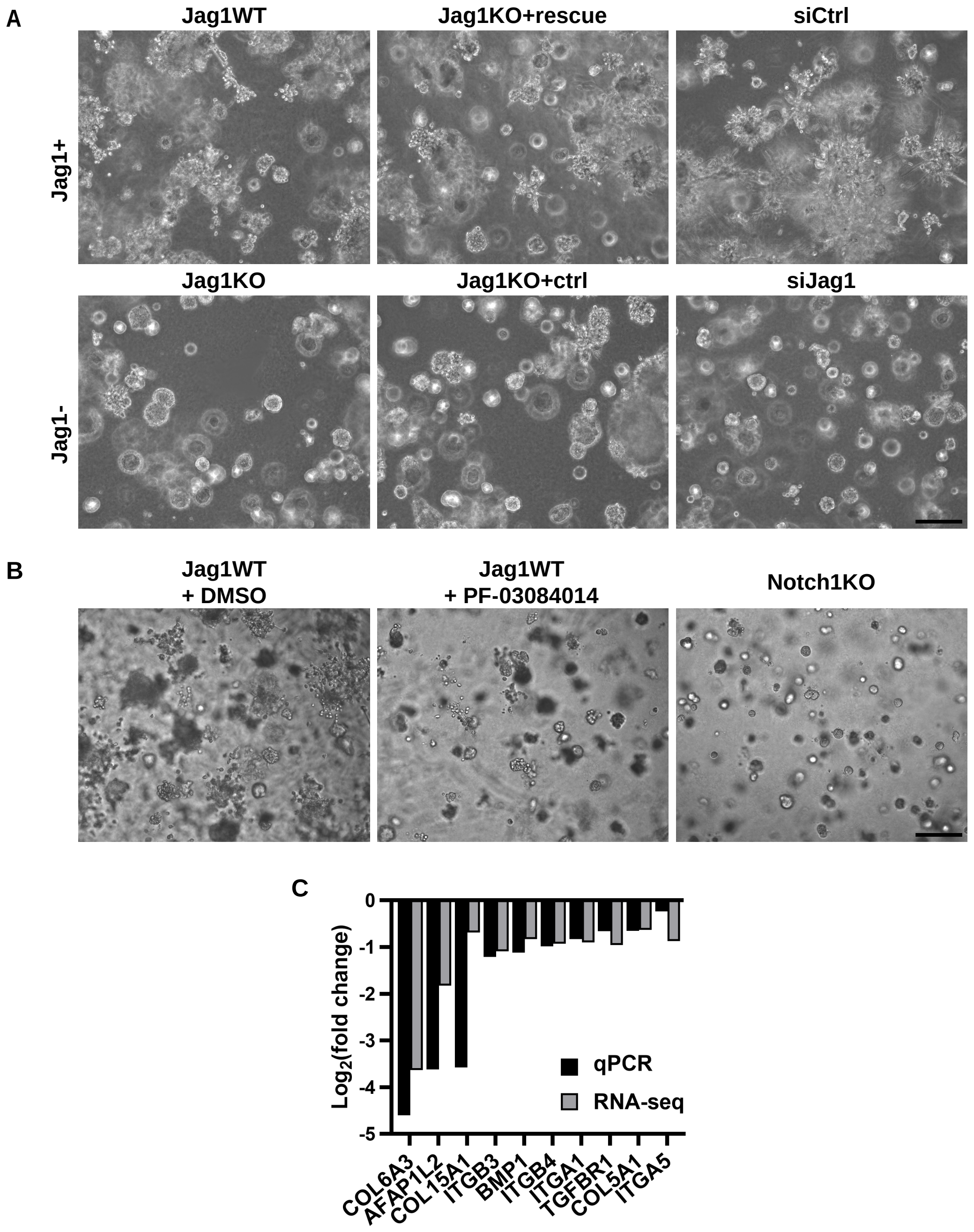

Fig. S2. Modulation of Jagged1-Notch signaling affects breast cancer spheroid growth and invasion. Representative spheroid images of (**A**) MDA-MB-231 Jag1WT and Jag1KO cells, Jag1KO cells transfected with Jag1-containing plasmid (Jag1KO+rescue) or empty vector plasmid (Jag1KO+ctrl), and Jag1WT cells transfected with non-targeting siRNA (siCtrl) or Jag1-targeting siRNA (siJag1), and (**B**) Jag1WT cells with inhibited Notch signaling activity either by a gamma-secretase inhibitor (PF-03084014) or knockout of Notch1 (Notch1KO). Scale bar 200 µm. (**C**) Log2 fold changes of genes downregulated in Jag1KO cells, determined by quantitative PCR (qPCR) or whole transcriptome sequencing (RNA-seq). Data are presented as mean of three independent experiments.

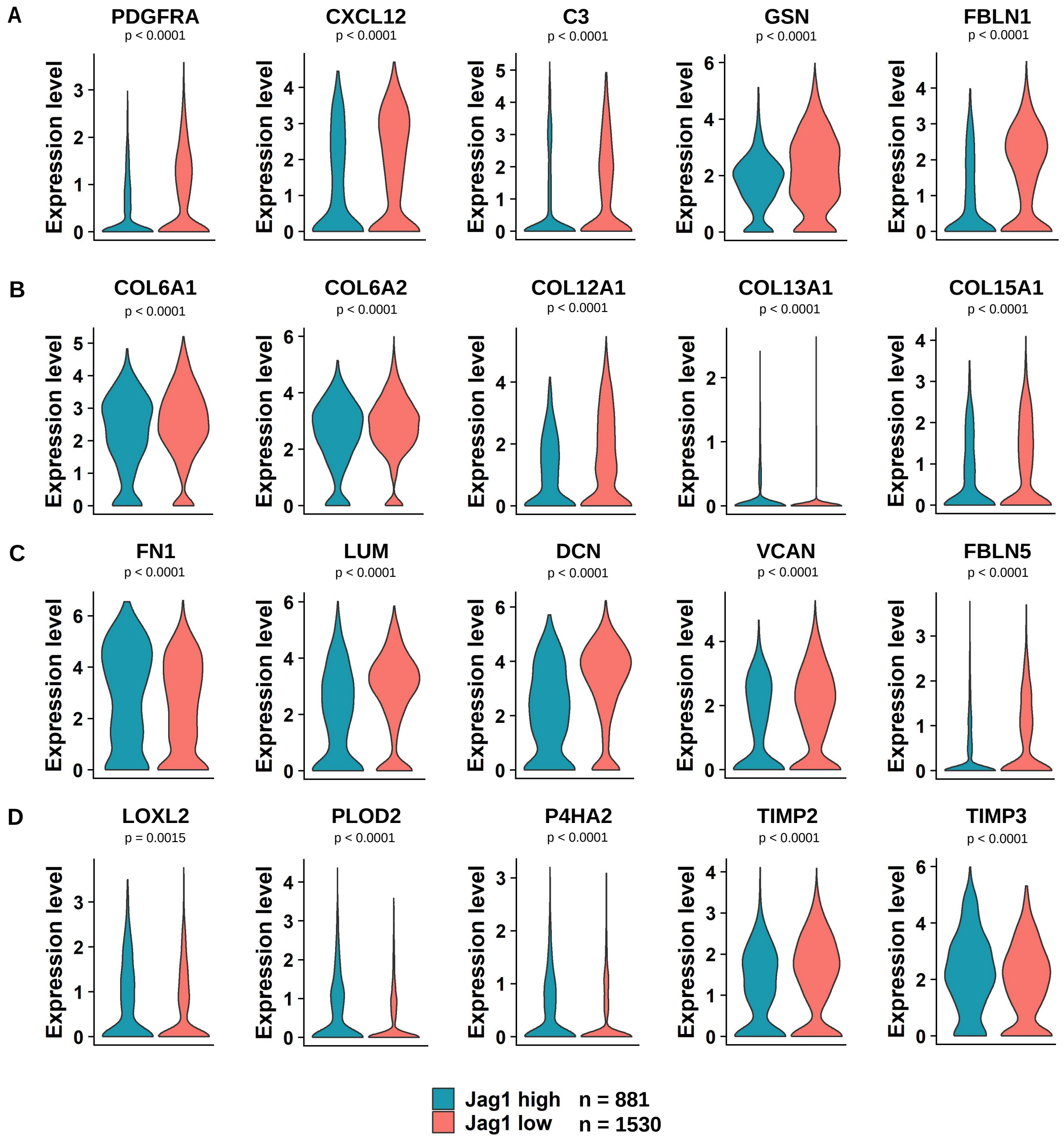

Fig. S3. Jagged1 expressed by cancer cells regulates expression of ECM and ECM remodeling genes in fibroblasts of the same tumors. Expression of (**A**) iCAF markers, (**B**) collagens, (**C**) ECM genes, and (**D**) ECM remodeling enzymes in scRNA-seq data (25) of fibroblasts from TNBC tumors with either high or low expression of Jagged1 in cancer cells. n = 881 of Jag1 high fibroblasts and n = 1530 of Jag1 low fibroblasts. p-values are calculated by Wilcoxon rank sum test with Benjamini-Hochberg adjustment.

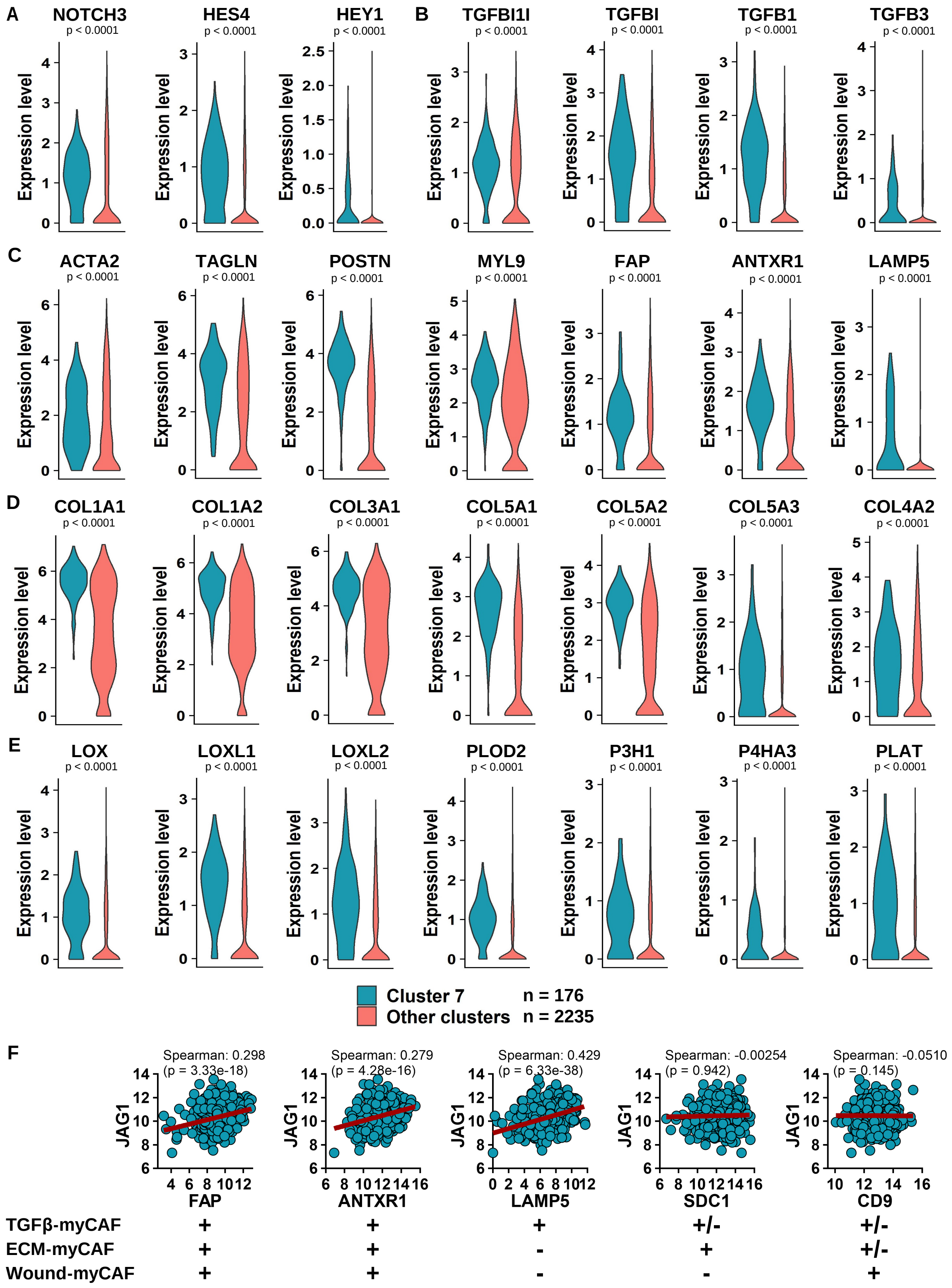

A
NOTCH3
p < 0.0001
HES4
p < 0.0001
HEY1
p < 0.0001
B
TGFBI1I
p < 0.0001
TGFBI
p < 0.0001
TGFB1
p < 0.0001
TGFB3
p < 0.0001
C
ACTA2
p < 0.0001
TAGLN
p < 0.0001
POSTN
p < 0.0001
MYL9
p < 0.0001
FAP
p < 0.0001
ANTXR1
p < 0.0001
LAMP5
p < 0.0001
D
COL1A1
p < 0.0001
COL1A2
p < 0.0001
COL3A1
p < 0.0001
COL5A1
p < 0.0001
COL5A2
p < 0.0001
COL5A3
p < 0.0001
COL4A2
p < 0.0001
E
LOX
p < 0.0001
LOXL1
p < 0.0001
LOXL2
p < 0.0001
PLOD2
p < 0.0001
P3H1
p < 0.0001
P4HA3
p < 0.0001
PLAT
p < 0.0001
Expression level
Cluster 7 n = 176
Other clusters n = 2235
F
Spearman: 0.298 (p = 3.33e-18)
Spearman: 0.279 (p = 4.28e-16)
Spearman: 0.429 (p = 6.33e-38)
Spearman: -0.00254 (p = 0.942)
Spearman: -0.0510 (p = 0.145)
JAG1
FAP
ANTXR1
LAMP5
SDC1
CD9
TGFβ-myCAF + + + +/- +/-
ECM-myCAF + + - + +/-
Wound-myCAF + + - - +

Fig. S4. High Jagged1 expression in cancer cells promotes differentiation of fibroblasts into a specific subtype marked by high Notch, TGFβ, and myCAF gene signatures. Expression of (**A**) Notch, (**B**) TGFβ, (**C**) CAF marker, (**D**) collagen, and (**E**) ECM remodeling enzyme genes in fibroblast cluster 7 in scRNA-seq data (25) of TNBC tumors. n = 176 of cluster 7 and n = 2235 of other cluster fibroblasts. p-values are calculated by Wilcoxon rank sum test with Benjamini-Hochberg adjustment. (**F**) Correlation of *JAG1* expression with markers of different myCAF subpopulations in a breast cancer patient mRNA expression dataset (TCGA, Cell 2015, n = 817) (26).

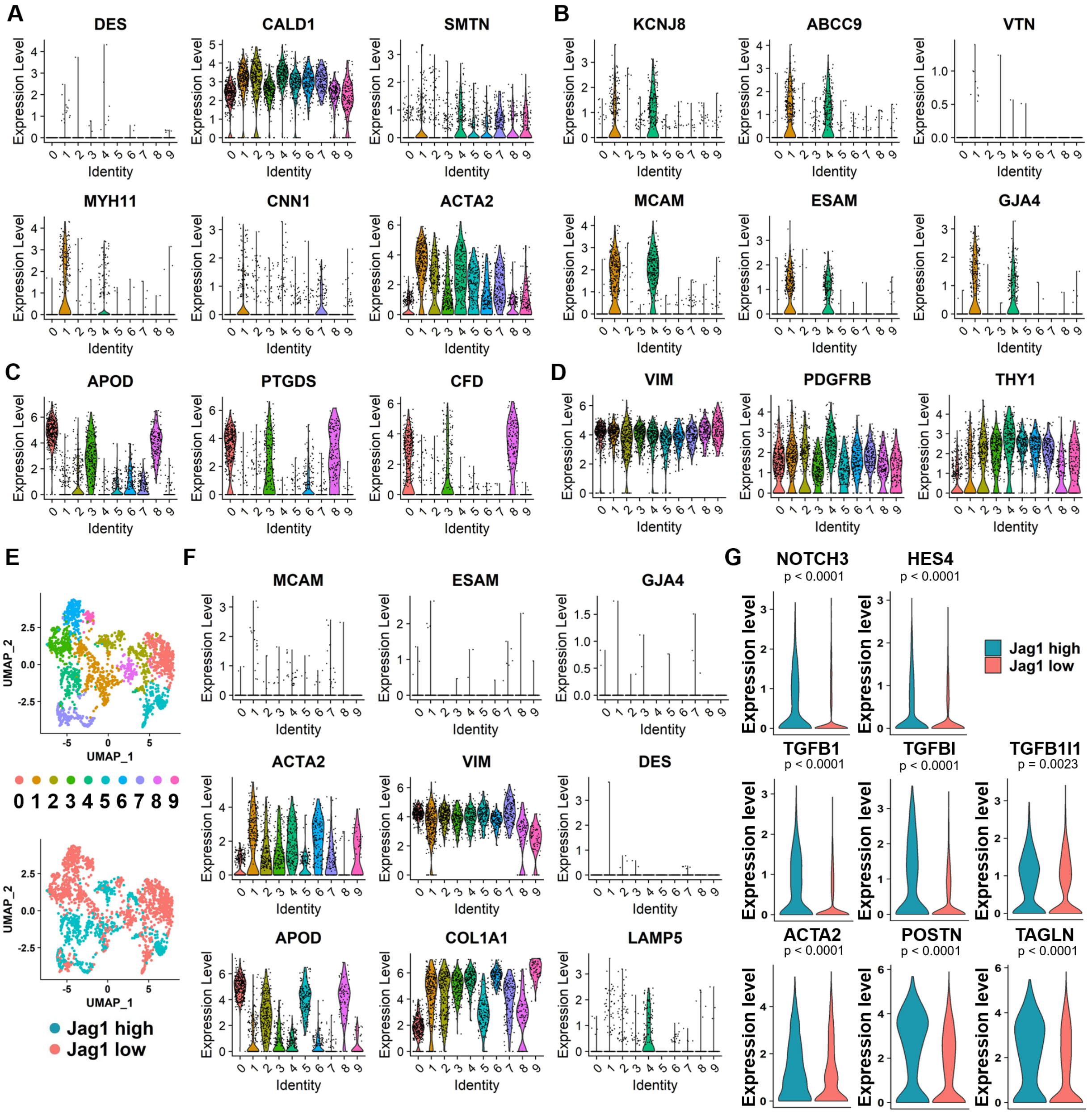

Fig. S5. Jagged1 induces myofibroblast differentiation in the scRNA-seq fibroblast cell population. Expression levels of (**A**) smooth muscle cell, (**B**) pericyte, (**C**) adipocyte, and (**D**) fibroblast markers in fibroblast clusters of the scRNA-seq data (25). (**E**) Re-clustered fibroblasts after removal of pericyte marker positive clusters. (**F**) Marker gene expression in fibroblast clusters from (**E**). (**G**) Notch, TGFβ, and myCAF gene expression in re-analyzed fibroblasts from (**E**) from either Jag1 high or Jag1 low tumors. p-values are calculated by Wilcoxon rank sum test with Benjamini-Hochberg adjustment.

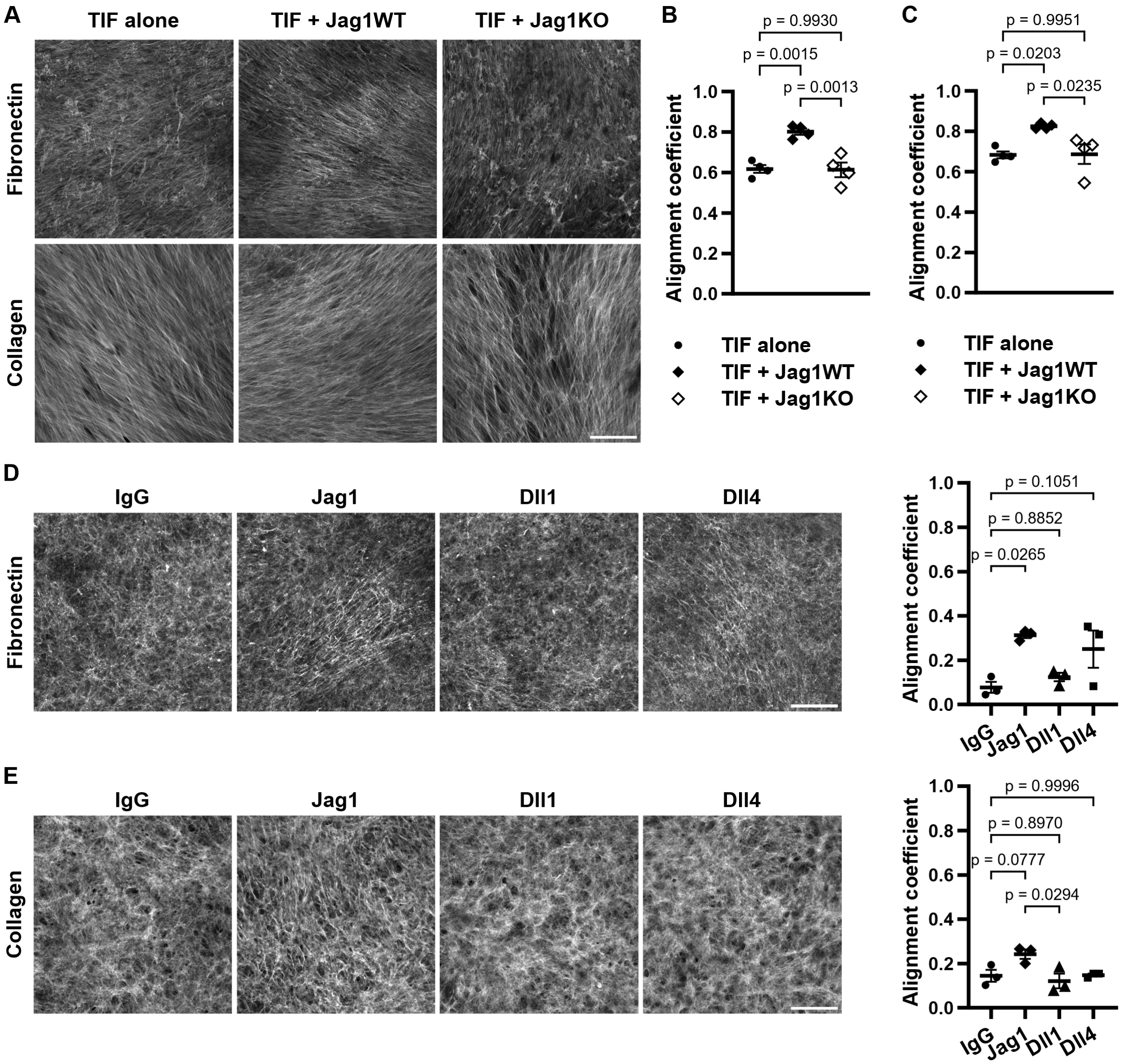

Fig. S6. Jagged1 directs extracellular matrix fiber alignment in cancer cell co-cultures with human fibroblasts and as a recombinant protein in fibroblast monocultures. (**A**) Representative images of fibronectin and collagen stainings of cell-derived matrices from human telomerase immortalized fibroblast (TIF) monocultures or TIF co-cultures with MDA-MB-231 Jag1WT or Jag1KO cells, and alignment coefficients of (**B**) fibronectin and (**C**) collagen fibers. Data are presented as mean ± SEM of four independent experiments. p-values are calculated by one-way ANOVA with Tukey's multiple comparisons test. Scale bar 100 μm. Representative images and fiber alignment coefficients for (**D**) fibronectin and (**E**) collagen from cell-derived matrices of MEF cells cultured on recombinant IgG, Jag1, Dll1, or Dll4 coated coverslips. p-values are calculated by one-way ANOVA with Tukey's multiple comparisons test. Data are presented as mean ± SEM of three independent experiments. Scale bar 100 μm.

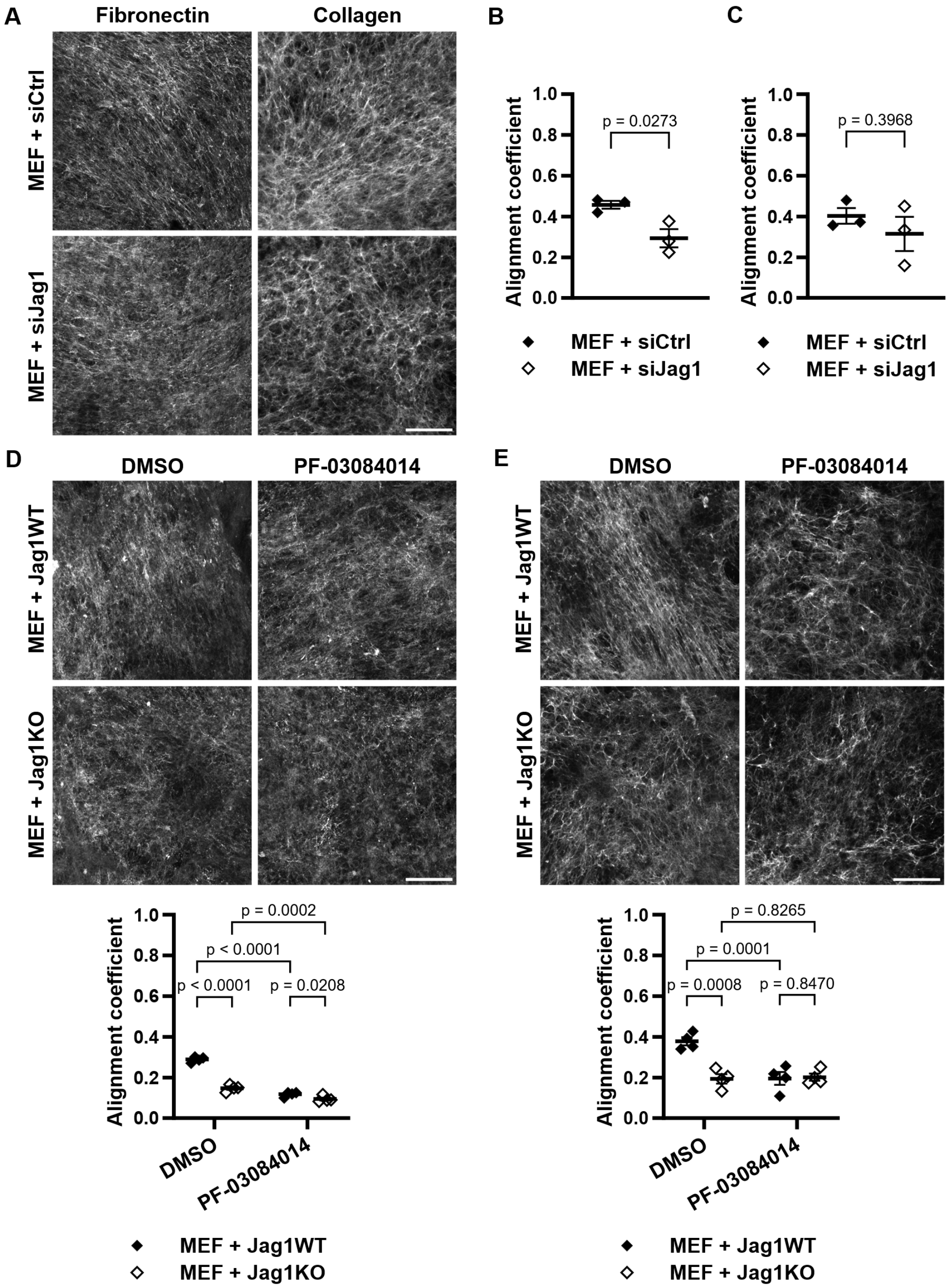

Fig. S7. Jagged1 expressed by cancer cells directs extracellular matrix fiber alignment via activation of Notch signaling. (**A**) Representative images of fibronectin and collagen stainings of cell-derived matrices (CDM) from MEF co-cultures with MDA-MB-436 cells transfected with either non-targeting siRNA (siCtrl) or Jag1-targeting siRNA (siJag1), and alignment coefficients of (**B**) fibronectin and (**C**) collagen fibers. p-values are calculated by two-tailed unpaired t-test. Data are presented as mean ± SEM of three independent experiments. Representative images and fiber alignment coefficients of (**D**) fibronectin and (**E**) collagen of CDMs from DMSO or PF-03084014 gamma-secretase inhibitor treated MDA-MB-231 Jag1WT or Jag1KO co-cultures with MEF cells. p-values are calculated by two-way ANOVA. Data are presented as mean ± SEM of four independent experiments. Scale bar 100 μm.

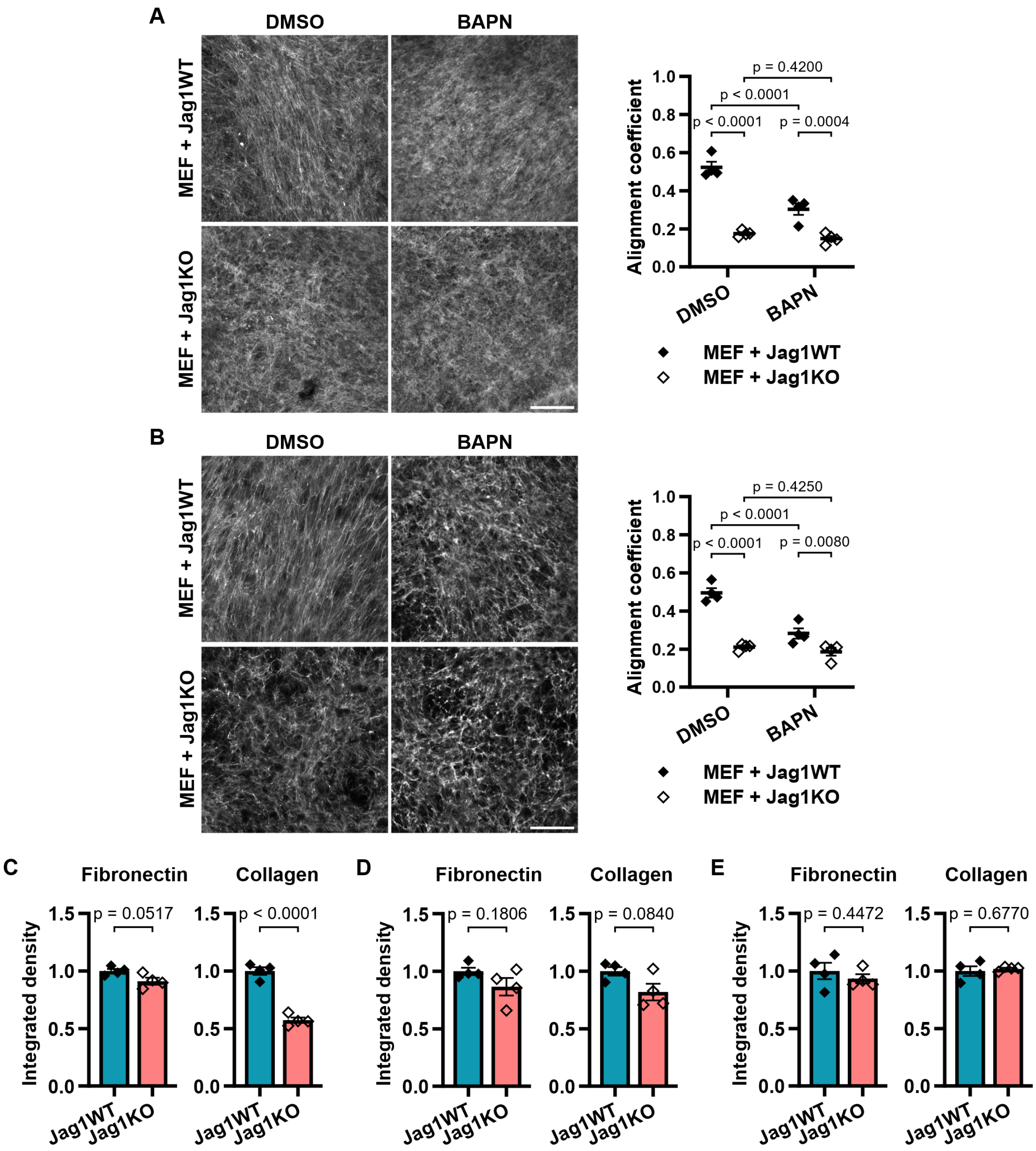

Fig. S8. Lysyl oxidase inhibition partly abrogates the induction of extracellular matrix fiber alignment by Jagged1. Representative images and fiber alignment coefficients for (**A**) fibronectin and (**B**) collagen from cell-derived matrices of MEF co-cultures with MDA-MB-231 Jag1WT or Jag1KO cells treated with either DMSO or BAPN (β-aminopropionitrile), a lysyl oxidase inhibitor. p-values are calculated by two-way ANOVA. Data are presented as mean ± SEM of four independent experiments. Scale bar 100 μm. Normalized integrated fluorescence densities of (**C**) control, (**D**) SB-431542 TGFβ inhibitor, and (**E**) recombinant TGFβ1 treated MEF co-culture CDMs. Data are presented as mean ± SEM of four independent experiments. p-values are calculated by two-tailed unpaired t-test with Welch's correction.

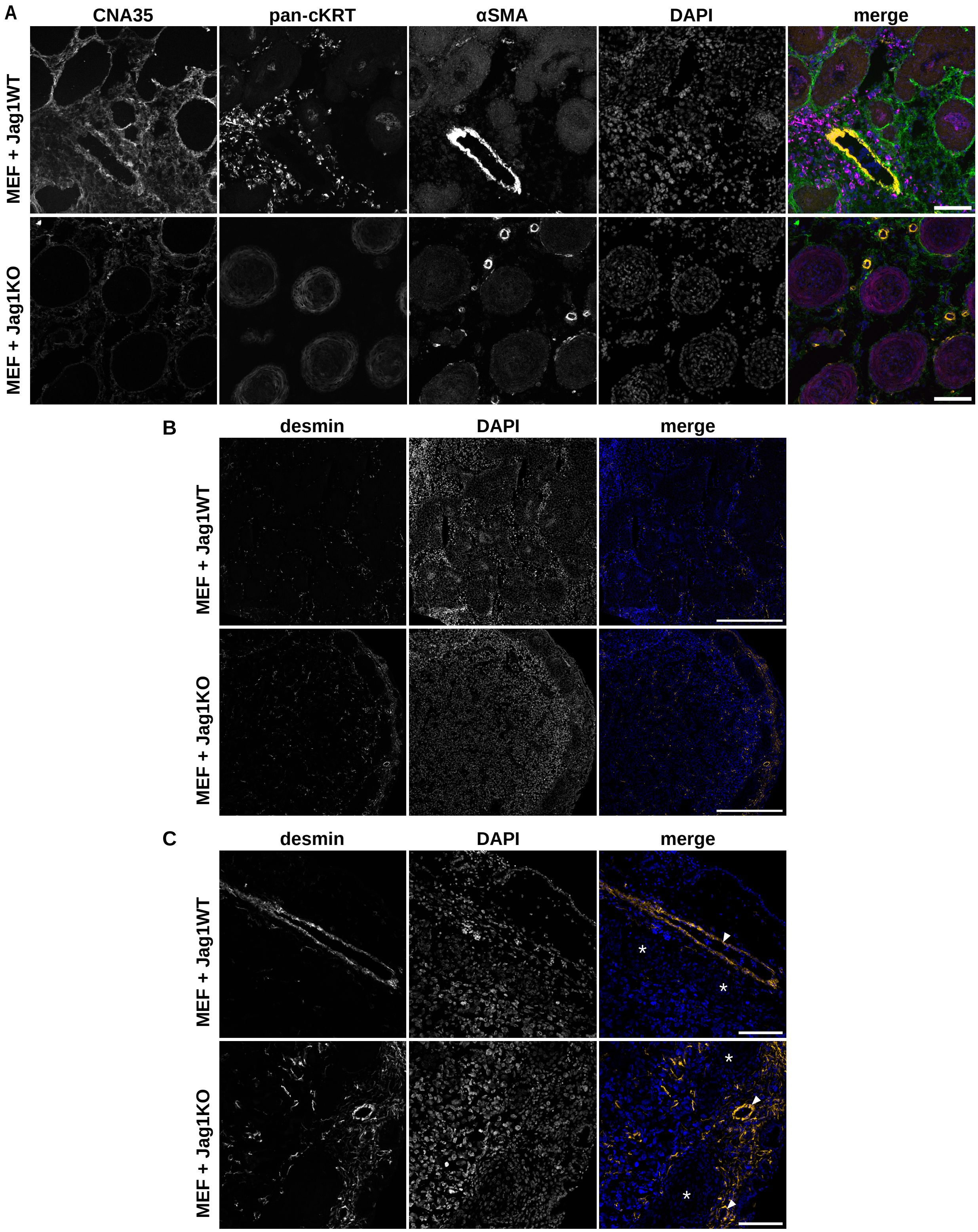

Fig. S9. Fibroblasts residing in glandular structures within *in vivo* CAM tumors express more αSMA and collagen when in contact with Jagged1-expressing cancer cells. (**A**) Higher magnification images of CAM tumor cryosections from figure 7E, stained for collagen (CNA35;

green), epithelial marker pan-cytokeratin (pan-cKRT; magenta), myofibroblast and smooth muscle cell marker αSMA (orange), and nuclei (DAPI; blue). Scale bar 100 μm. (**B**) CAM tumor cryosections stained for smooth muscle cell marker desmin (orange) and nuclei (DAPI; blue). Scale bar 500 μm. (**C**) Higher magnification images of (**B**). White stars in the merge images denote the glandular structures and white arrow heads denote blood vessels. Scale bar 100 μm.

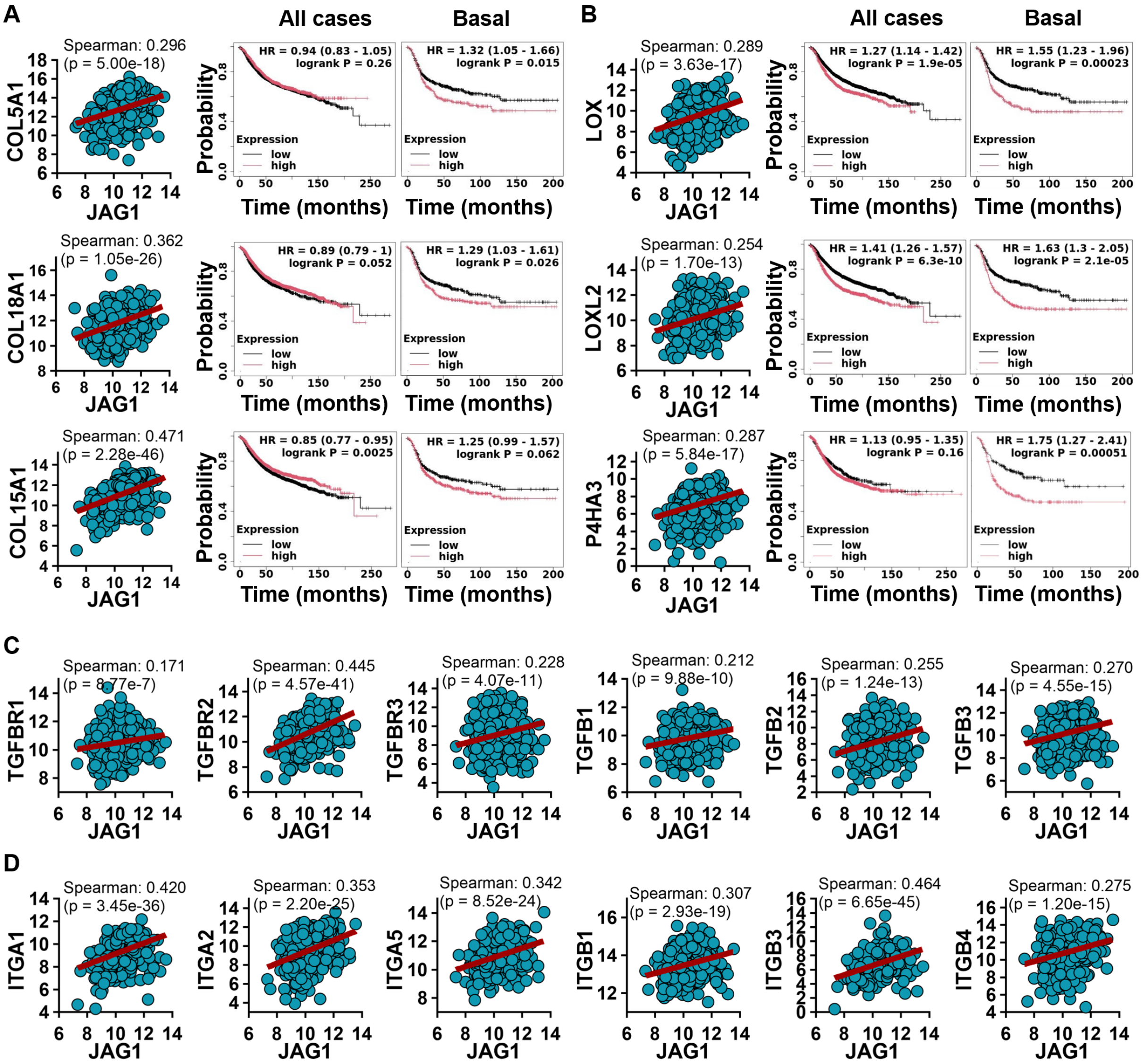

Fig. S10. Jagged1 expression correlates with ECM, TGFβ, and integrin gene expression in breast cancer patient data. mRNA expression level correlation with Jag1 mRNA expression in breast cancer patients (TCGA, Cell 2015, n = 817) (26) and relapse-free survival of patients with low (black) or high (red) expression of (**A**) collagens *COL5A1*, *COL18A1*, and *COL15A1* and (**B**) extracellular matrix modifying enzymes *LOX*, *LOXL2*, and *P4HA3* in all breast cancer cases and in basal breast cancer separately in the Kaplan-Meier Plotter database (58). mRNA expression level correlation with Jag1 mRNA expression in breast cancer patients (TCGA, Cell 2015, n = 817) (26) of (**C**) TGFβ receptors and ligands *TGFBR1*, *TGFBR2*, *TGFBR3*, *TGFB1*, *TGFB2*, and *TGFB3* and (**D**) integrins *ITGA1*, *ITGA2*, *ITGA5*, *ITGB1*, *ITGB3*, and *ITGB4*.

Table S8. Primers used for quantitative PCR.

Gene	Forward primer	Reverse primer
COL6A3	CGTGCTGTCTCCAGCGTTTA	CACCACGTCCTTTTCACCTG
AFAP1L2	ATCCAGACGGAACAGCAGAG	TGTGTACCGCTTCACCTCAG
COL15A1	GCCACGGAGGTCAGTTCAAT	AGTTATCCACAAGGCGGACG
ITGB3	TGCGAGTGTGACGACTTCTC	GTCCAGTCGGAGTCACACAG
BMP1	ACGCTCGGAACACATTCTCC	CACTTGTAAAGCTTGCGGGC
ITGB4	CCAGCTCAGCAACCCTAAGT	GAAGCTCCGGTCCAGTTCAT
ITGA1	CAATGACTTTCAGCGGCCC	GGCCAACTAACGGAGAACCAA
TGFBR1	GGTTCCGTGAGGCAGAGATTT	TCTGACACCAACCAGAGCTGA
COL5A1	TACGAAGACCCCGAAGACCT	CCCTTCACTGGTTTCAGGCA
ITGA5	TGGCCTTCGGTTTACAGTCC	GGAGAGCCGAAAGGAAACCA
JAG1	AATGGCTACCGGTGTGTCTG	CCCATGGTGATGCAAGGTCT
UBC	CACTTGGTCCTGCGCTTGA	CAATTGGGAATGCAACTTTAT

Table S1. Genes differentially expressed between Jag1 high and Jag1 low TNBC cells in the scRNA-seq data.

Table S2. Genes differentially expressed between MDA-MB-231 Jag1WT and Jag1KO, Jag1KO+rescue and Jag1KO+ctrl, and siCtrl and siJag1 cells.

Table S3. Genes correlating with JAG1 in breast invasive carcinoma (TCGA, Cell 2015) patient data.

Table S4. Genes differentially expressed between Jag1 high and Jag1 low fibroblasts in the scRNA-seq data.

Table S5. Genes differentially expressed between cluster 7 and other fibroblasts in the scRNA-seq data.

Table S6. Genes differentially expressed between Jag1 high and Jag1 low re-analyzed fibroblasts after removal of pericyte marker positive clusters in the scRNA-seq data.

Table S7. Genes differentially expressed between cluster 4 and other re-analyzed fibroblasts after removal of pericyte marker positive clusters in the scRNA-seq data.

Data file S1: R code used for scRNA-seq data analysis.

Data file S2: Statistical source data.

Unprocessed western blots

Figure 4D

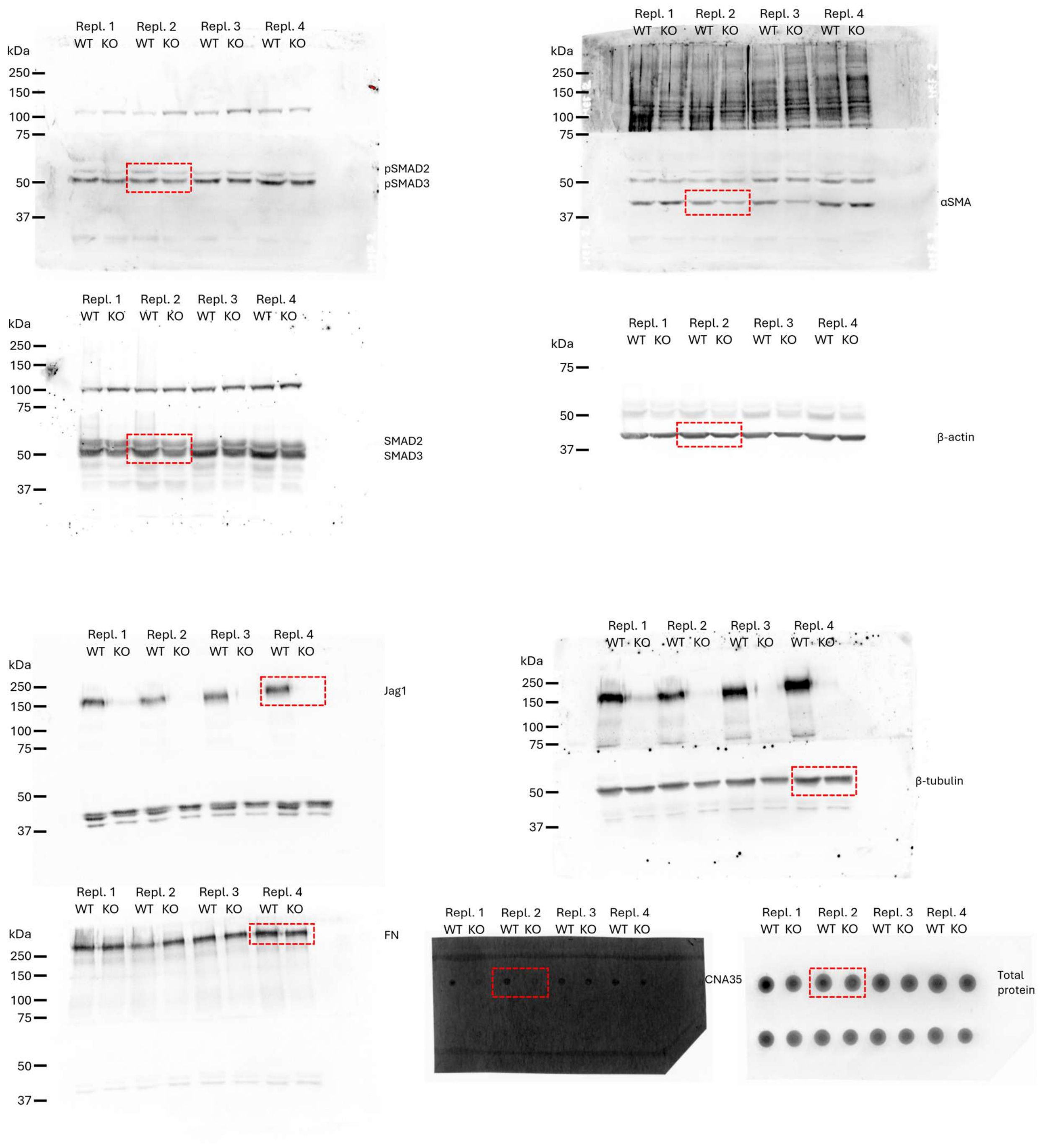

Figure 4E

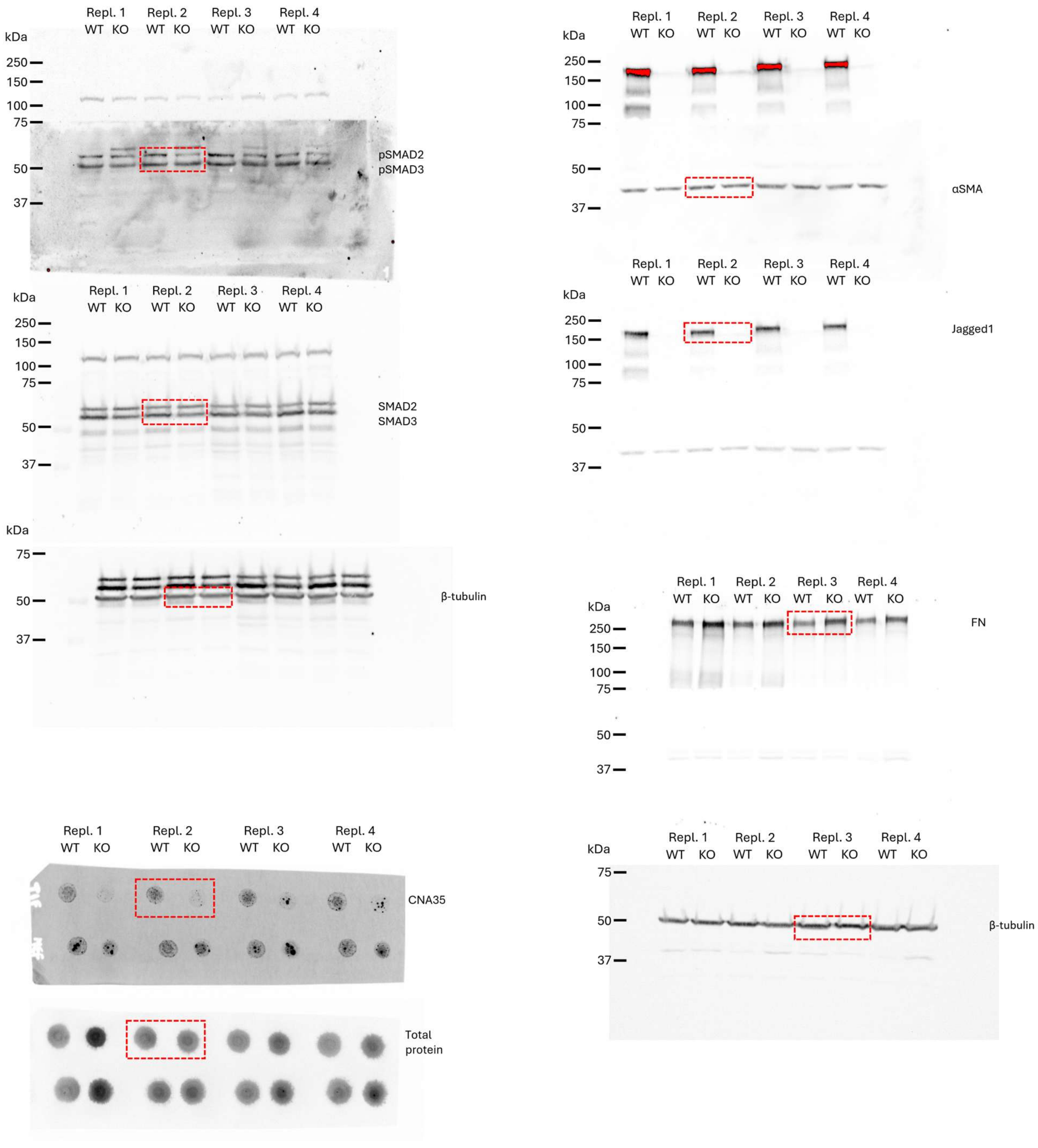
Repl. 1 Repl. 2 Repl. 3 Repl. 4
WT KO WT KO WT KO WT KO
kDa
250
150
100
75
50
37
pSMAD2
pSMAD3
SMAD2
SMAD3
β-tubulin
αSMA
Jagged1
FN
CNA35
Total protein

Figure 6B

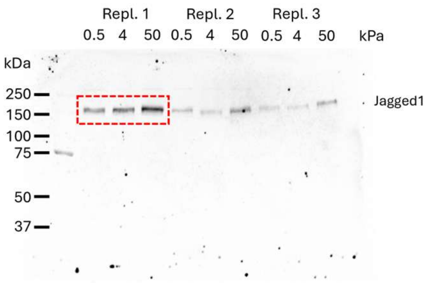

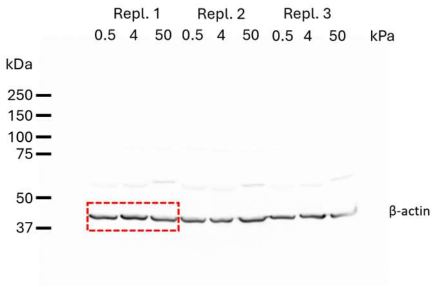

Figure 6C

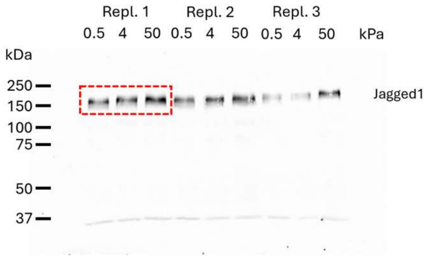

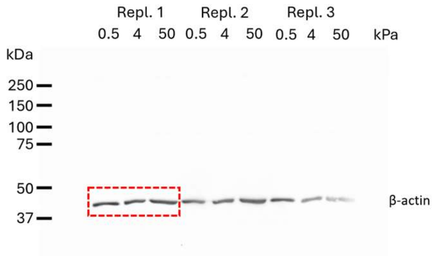

Figure S1A

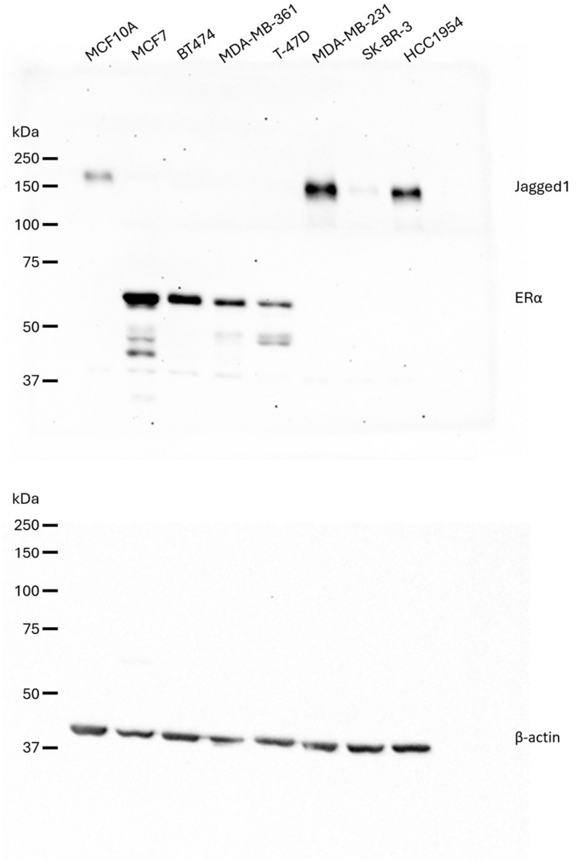
MCF10A
MCF7
BT474
MDA-MB-361
T-47D
MDA-MB-231
SK-BR-3
HCC1954
kDa
250
150
100
75
50
37
Jagged1
ERα
kDa
250
150
100
75
50
37
β-actin
